# Protocol for high-quality RNA sequencing, cell surface protein analysis, and genotyping in single cells using TARGET-seq+

**DOI:** 10.1016/j.xpro.2025.103832

**Published:** 2025-05-21

**Authors:** N. Asger Jakobsen, Sven Turkalj, Paresh Vyas

**Affiliations:** 1 MRC Molecular Haematology Unit, MRC Weatherall Institute of Molecular Medicine, University of Oxford, Oxford, UK; 2 Oxford Centre for Haematology, Oxford University Hospitals NHS Foundation Trust, Oxford, UK; 3 Department of Haematology, Oxford University Hospitals NHS Foundation Trust, Oxford, UK

**Keywords:** Flow Cytometry, Gene Expression, Genomics, Molecular Biology, RNA-seq, Sequencing, Single Cell, Stem Cells

## Abstract

Studying the consequences of somatic mutations in pre-malignant and cancerous tissues is challenging due to noise in single-cell transcriptome data and difficulty in identifying the clonal identity of single cells. We optimized TARGET-seq to develop TARGET-seq+, which combines RNA sequencing (RNA-seq), the analysis of cell surface protein expression, and genotyping in single cells with improved sensitivity. We describe the steps for cell isolation, the preparation of single-cell RNA-seq (scRNA-seq) and genotyping libraries, and sequencing. We also provide guidance on the analysis of single-cell genotyping, transcriptome pre-processing, and data integration.

For complete details on the use and execution of this protocol, please refer to Jakobsen et al.^1^

## Highlights

• Improved scRNA-seq with high-sensitivity genotyping and index sorting of single cells
• Study the transcriptional consequences of somatic mutations in primary samples
• Guidance on barcoding, high-throughput automation, and computational analysis

## Abstract (editor highlights)

Publisher’s note: Undertaking any experimental protocol requires adherence to local institutional guidelines for laboratory safety and ethics.

## Graphical abstract

**Figure:**
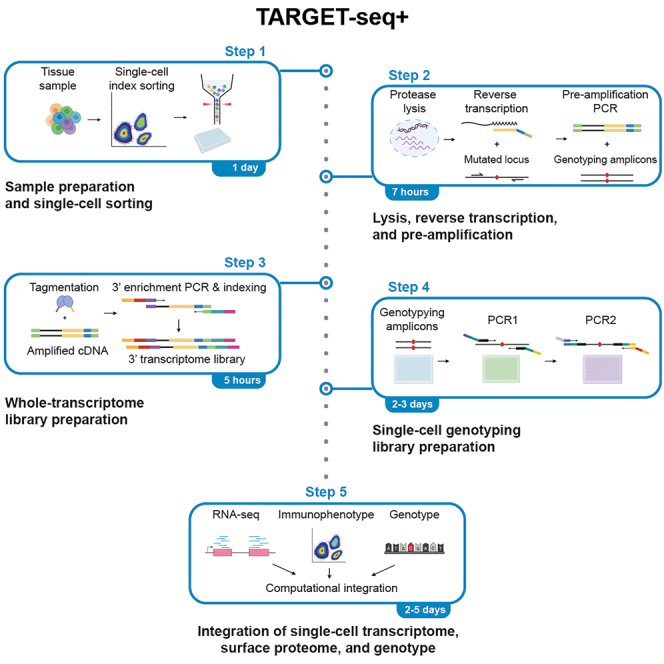

## Before you begin

TARGET-seq^2^^,^^3^ is a plate-based method for simultaneous capture of genotype, gene expression, and surface protein expression from single cells, enabling gene expression and immunophenotype to be compared between genetic clones within the same tissue. Compared to droplet-based methods, TARGET-seq provides high sensitivity mutational detection with low allelic dropout rates, which is particularly important when profiling rare cell types and complex clonal hierarchies. We further optimized the method to develop TARGET-seq+, which we describe in this protocol. Both TARGET-seq and TARGET-seq+ capture cell genotype by the addition of targeted primers to the RT-PCR reaction which amplify gDNA and cDNA loci harboring mutations of interest in parallel with whole transcriptome amplification. The original TARGET-seq protocol relies on a modified version of Smart-seq2^4^^,^^5^ for cDNA library construction. More recently, Smart-seq3^6^ has been developed, with substantially improved sensitivity. In developing TARGET-seq+,^1^ we incorporated elements of the Smart-seq3 chemistry into the TARGET-seq protocol. As a result, TARGET-seq+ detects more genes per cell, with improved detection of lowly expressed genes, reduced transcript dropouts, and yields a higher proportion of cells passing quality control (QC) while maintaining the very high accuracy and efficiency of single-cell genotyping. Compared to the original protocol, we also modified the transcriptome library indexing to incorporate i5 plate indexes in the 3′ cDNA library. This introduces redundancy between the i7 and i5 plate indexes which mitigates against read misassignment resulting from index hopping. This is particularly important when using instruments with patterned flow cells, such as the NovaSeq platform. Finally, we optimized the automation strategy and reduced the reagent volumes at several steps, thus reducing the overall cost per cell.

The protocol below describes the specific steps for applying TARGET-seq+ to frozen primary human bone marrow (BM) or peripheral blood (PB) samples. Note that the steps 8–18 in part 2 may change depending on the tissue of interest, whilst parts 1, 3, 4, 5, and 6 should remain the same. Prior to the execution of TARGET-seq+ on the sample of interest, several pilot experiments need to be performed first to (a) define the optimal number of PCR cycles for the tissue of interest; and (b) to test genotyping primers. These pilot experiments, described in preparation 1, 2, 3, and 4, simulate the full protocol (step-by-step method details).

### Institutional permissions

Human samples were collected with informed consent under ethically approved protocols (NHS REC 17/YH/0382). Written informed consent was obtained in accordance with the Declaration of Helsinki. If TARGET-seq+ is performed on primary human samples, the study requires the approval by an Ethics Committee or by an equivalent organization before the collection of clinical biopsies. Informed consent must be collected from all patients.

### Preparation 1: Determine the optimal number of PCR cycles for the specific tissue type

**Timing: 2 days**

**Figure 1:**
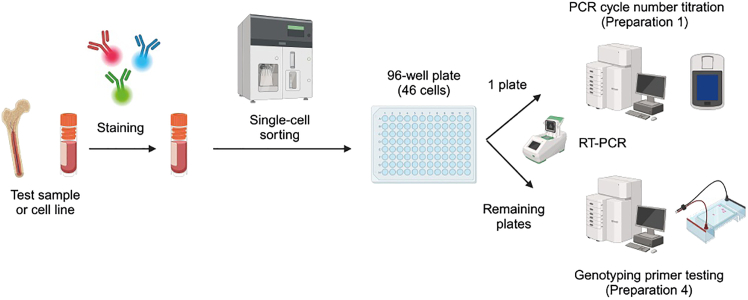
Pilot TARGET-seq+ experiment Prior to the execution of TARGET-seq+ on samples of interest, the user needs to define the optimal PCR cycle number for the tissue of interest (preparation 1) and test genotyping primers in single cells (preparation 4). The user can sort multiple 96-well plates in this pilot experiment; these plates will be used in preparation 1 and 4. Ideally, cells from a sample from the same tissue as the sample of interest should be used. RT-PCR, reverse transcription and PCR.

Prior to proceeding with primary human samples, we strongly recommend performing a pilot experiment to determine the optimal number of PCR cycles required for cDNA amplification (Figure 1). Due to differences in mRNA content, different cell types may require different amounts of amplification to obtain sufficient cDNA for downstream QC and library preparation.

Aim to generate 0.25–0.5 ng/μL per single cell after bead purification of cDNA libraries, and not more than 2 ng/μL. We typically use 19 cycles for JURKAT cells and 21 cycles for human hematopoietic stem and progenitor cells (HSPCs). The number of PCR cycles can be increased for smaller cells with low mRNA content or increased for large cells with high mRNA content.

***Note:*** We recommend preparing multiple plates of FACS-sorted single cells that can be used for pilot experiments. ∼3 plates will be used in preparation 1 for determining the optimal number of PCR cycles. The remaining plates (stored at −80°C) will be used in preparation 4 for testing genotyping primers in single cells (Figure 1).

**CRITICAL:** The preparation of lysis buffer plates and all steps up to PCR pre-amplification are critical for successful cDNA generation and should be performed in a pre-PCR clean area, ideally in a biosafety cabinet, to avoid contamination from PCR products and RNases. Clean the biosafety cabinet and pipettes with a cleaning agent to remove RNases (e.g., RNaseZap) and use RNase-free filter tips during the entire protocol.

1. Prepare generic lysis buffer mix as outlined in the table below:

**Table:** Lysis buffer

Reagent	Final concentration in the lysis buffer	Volume per well (μL)	Volume for 480 wells (in 96-well plates) + 15% dead volume (μL)
Nuclease-Free Water		3.91	2150.5
Poly-ethylene Glycol 8000 (40% solution)	6.7%	1	550
Triton X-100 (10% solution)	0.1%	0.06	33
RNase Inhibitor	0.5 U/μL	0.08	44
dNTPs (10 mM/each)	0.67 mM/each	0.4	220
Protease (1.09 AU/mL)	27 mAU/mL	0.15	82.5
Oligo(dT)-ISPCR	0.67 μM	0.4	220
**Total**		**6.0**	**3300**

2. Dispense 6 μL of lysis buffer into each well within alternate columns of a 96-well plate.

***Note:*** Ensure that the poly-ethylene glycol 8000 (PEG 8000) is fully mixed into solution, by mixing with water and pipetting up and down until the liquid is clear, before adding the remaining reagents.

***Note:*** This lysis buffer contains a generic oligo(dT)-ISPCR primer which has no single-cell barcode. This is used for testing and validation experiments, where sequencing is not required, but should not be used when performing experiments where single-cell RNA-seq libraries will be sequenced.

***Note:*** We recommend working in 96-well plates for pilot experiments, as this makes hand-pipetting easier and less error-prone. Leave alternate columns of the plate empty (i.e. fill only 48 wells on each plate). This makes it easier to dispense master mix into each column within a short time frame during the RT step. In these Preparation paragraphs, we will often refer to the step-by-step method details for detailed procedures; note, however, that the volumes for these pilot experiments should be doubled, given the use of 96-well over 384-well plates.

**Pause point:** You can store the sorting plates containing lysis buffer at −80°C for up to 1 month until the sort.

3. Sort single cells into plates with lysis buffer:
  a. Prepare a single-cell suspension from a control sample with the cell type of interest.
  b. Sort single cells using FACS into the plates with lysis buffer (point 2). Leave 2 wells per plate empty as no-template controls.
  c. Seal sorted plates with aluminum film and snap freeze on dry ice.

***Note:*** See part 2 for details.

***Note:*** We recommend sorting multiple 96-well plates with control single cells at this stage. These can be used for test experiments to validate genotyping primers in single cells (preparation 4).

**Pause point:** Sorted plates can be stored at −80°C for up to 3 months. Processing plates after 3 months is associated with higher rates of RNA degradation.

4. Perform RT-PCR pre-amplification steps as described in part 3, omitting target-specific genotyping primers.

***Note:*** We recommend initially testing at least 3 different PCR cycling conditions per cell type (e.g., for HSPCs: 19 cycles, 21 cycles, and 23 cycles of PCR amplification).

***Note:*** Double the volumes compared to part 3 when working in 96-well plates here.

5. After RT-PCR, dilute the amplified cDNA 1:2 and purify half of the volume from each well with AMPure XP beads as follows:
  a. Prior to starting, equilibrate the AMPure XP beads at 21°C for 30 min. Vortex thoroughly to ensure that the beads are fully mixed with the buffer.
  b. Prepare 80% ethanol solution in nuclease-free water.
  c. Dispense 12 μL of AMPure XP beads into each well of a V-bottom 96-well plate (equal to the number of wells in the RT-PCR plate to be purified).
  d. Dilute the amplified cDNA 1:2 by adding 20 μL nuclease-free water to each well of the RT-PCR plate, and pipette to mix.
  e. Transfer 20 μL of diluted cDNA from each well to the V-bottom 96-well plate containing AMPure XP beads (0.6:1 beads to cDNA ratio) and pipette to mix. Avoid bubbles. Keep each well separate to purify cDNA libraries from each cell separately. Incubate for 5 min or longer at 21°C.
  f. Place the plate on a 96-well magnetic stand and incubate for 2 min until the liquid is clear of beads. Carefully remove the supernatant with a P200 pipette.
  g. Wash the beads twice by adding 100 μL of 80% ethanol to each well, incubating for 30 sec and then removing and discarding the supernatant, taking care not to disturb the beads. After the second wash, carefully remove any residual ethanol using 20 μL tips.
  h. Let the beads air-dry for 2–4 min. The beads are dry enough when the surface of the pellet changes from shiny to matt. Be careful not to over-dry the beads as this makes it difficult to resuspend them and may reduce cDNA yield.
  i. Remove the plate from the magnet. Resuspend beads in 10 μL EB buffer and mix thoroughly by pipetting. Incubate for 5 min to elute cDNA.
  j. Place the plate back on the magnet and wait for 2–3 min for the supernatant to be completely clear of beads. Transfer the supernatant containing purified cDNA to a new plate.

**Pause point:** The purified cDNA can be stored at −20°C for at least one year.

**Figure 2:**
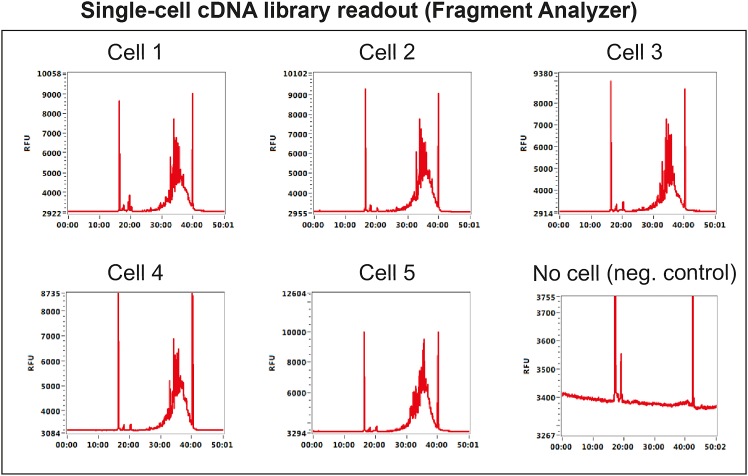
Optimal cDNA libraries from single cells Fragment analyzer traces showing successful cDNA libraries derived from 5 single cells. As negative control, an empty well is used.
  b. Quantify the cDNA concentration per cell using Qubit. Optimal PCR conditions will generate 0.25–0.5 ng/μL cDNA per single cell after bead purification and not more than 2 ng/μL.

6. Perform quality control on the cDNA:
  a. Check cDNA quality by capillary electrophoresis, such as using a Bioanalyzer (Agilent), Fragment Analyzer (Agilent) or TapeStation (Agilent). Optimal cDNA traces are shown in Figure 2.

### Preparation 2: Design and validate target-specific pre-amplification genotyping primers

**Timing: 2 days**

**Figure 3:**
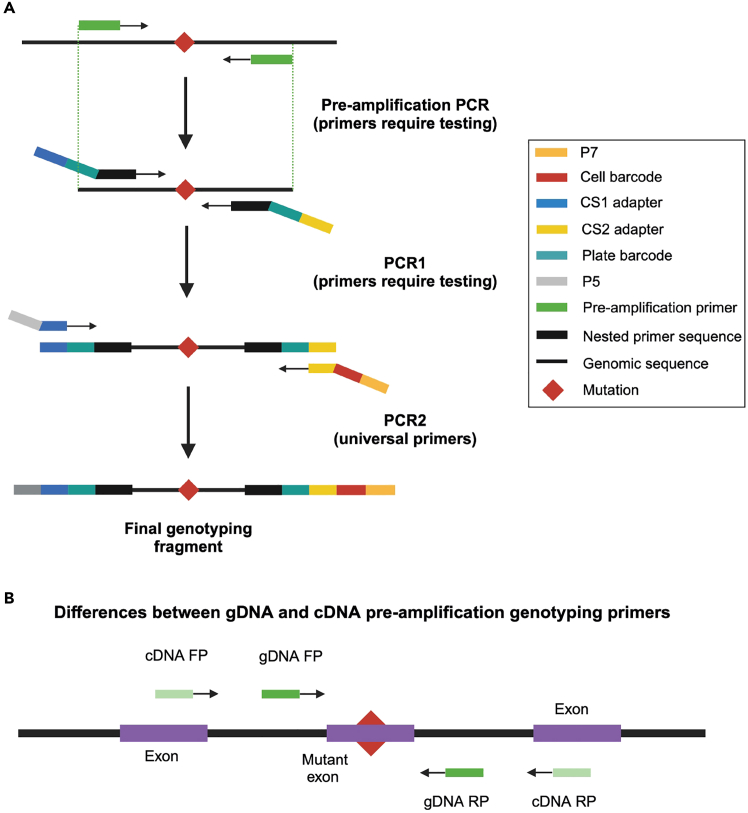
Genotyping primers (A) Schematics of primers used for single-cell genotyping. Sequence color-coding on the right. In the pilot experiments, both pre-amplification and nested PCR1 primers need to be tested. (B) If both gDNA and cDNA pre-amplification genotyping primers are used, gDNA primers need to anneal fully or partially to intronic sequences, whilst cDNA primers must anneal to exons outside of the exon harboring the mutation.

The key advantage of TARGET-seq+ over classical scRNA-seq approaches is the simultaneous capture of mutational status from single cells, enabling gene expression to be compared between genetic clones.^1^^,^^2^^,^^3^^,^^7^ This is achieved via the addition of target-specific genotyping primers enriching for mutant loci of interest. Briefly, the ‘pre-amplification’ genotyping primers, which are added to the RT-PCR, enrich for the mutant loci from gDNA and, optionally, from cDNA, in parallel with cDNA amplification. The pre-amplified genotyping fragments are then further enriched and fully barcoded in two consecutive PCR steps^2^^,^^3^^,^^8^^,^^9^ (Figure 3A). PCR1 uses target-specific primers nested within the initial amplicon, which add a plate barcode and universal CS1/CS2 adapters. These adapters are bound by universal primers in PCR2, which then attach a cell barcode and P5/7 sequencing adapters, yielding a barcoded fragment ready for sequencing. For single-cell genotyping to work optimally, both the pre-amplification and the nested genotyping primers need to be tested for efficient amplification. Primer validation is performed first on bulk gDNA/cDNA (preparation 2 and 3) and then in single cells.

***Note:*** A list of previously validated pre-amplification and nested genotyping primers that we used in our prior studies^1^^,^^10^ is provided in Table S1. One or two of these primer pairs can also be used as positive control for bulk testing.

**CRITICAL:** All genotyping primers not previously used in TARGET-seq+ should be validated according to instructions in preparation 2, 3, and 4. Do not assume that primers would work efficiently in TARGET-seq+ if they worked successfully in other protocols (different enzymes/PCR conditions). Even for primers validated for the original TARGET-seq method,^2^^,^^7^ we recommend re-testing with TARGET-seq+ conditions, due to the differences in the pre-amplification chemistry.

7. Design gDNA pre-amplification genotyping primer pairs for each locus using Primer3,^11^ Primer Blast,^12^ or another tool for primer design. Design at least 2 pairs per locus.
  a. Use the following criteria:
    i. Aim for an amplicon size 200–900 bp, if possible.
    ii. Aim for a primer length 19–25 bp.
    iii. Melting temperature should be 57°C–63°C; GC content should be 20%–80%.
    iv. Concentration of divalent cations: 3.5.
    v. Check for primer specificity against a genomic and transcriptomic reference, using Primer Blast. If possible, select pairs that show no/minimal non-specific binding.
    vi. If possible, design the primers to anneal to the introns adjacent to the mutant exon, which confers specificity for gDNA over cDNA. The primers can also anneal to intron-exon junctions. This is required if you want to use both gDNA and cDNA genotyping primers (Figure 3B).
  b. Order the primers and reconstitute them at 100 μM in TE buffer (10 mM Tris-HCl pH 8.0, 0.1 mM EDTA).
  c. Prepare 4 μM primer dilutions in nuclease-free water to be used in preparation 2, point 10.

***Note:*** If the mutant exon is long (typically > 700 bp), it may not be possible to use separate gDNA and cDNA genotyping primers. In that case, design primer pairs annealing to the mutant exon, which will serve for amplification from both gDNA and cDNA. Also, if > 4 loci are genotyped, we recommend using only gDNA genotyping primers (in that case, skip the next point).

8. Design cDNA pre-amplification genotyping primer pairs for each locus using Primer3, Primer Blast, or another tool for primer design. Design at least 2 pairs per locus.
  a. Use the following criteria:
    i. Same as criteria in points 7ai-7aivi for gDNA primers, checking for specificity only against a transcriptomic reference.
    ii. If possible, design the primers to anneal to the exons adjacent to the mutant exon, which confers specificity for cDNA over gDNA (Figure 3B). The primers can also anneal to the junction between two exons. This is required if you want to use both gDNA and cDNA genotyping primers.
  b. Order the primers and reconstitute them at 100 μM in TE buffer.
  c. Prepare 4 μM primer dilutions in nuclease-free water to be used in preparation 2, point 10.

***Note:*** From our experience, while gDNA genotyping primers are mandatory, cDNA genotyping primers are optional. While cDNA genotyping primers slightly improve mutation detection (by ∼5%) and correct for allelic drop-out (ADO) of the gDNA amplicon in a small proportion of cells, most cells are successfully genotyped even if only gDNA primers are used. If 4 or more loci are genotyped, it may be best to use exclusively gDNA primers, to avoid overloading the RT-PCR with genotyping primers.

9. Prepare a mock 2× RT buffer (to simulate RT-PCR conditions) for 750 reactions:

**Table:** 2× mock RT buffer (stored at −20°C for a maximum of two months)

Reagent	1 reaction	750 reactions
Poly-ethylene Glycol 8000 (40%)	0.5 μL	375 μL
Triton X-100 (10% solution)	0.03 μL	22.5 μL
Tris-HCl pH 8.3 1 M	0.1 μL	75 μL
NaCl 1 M	0.12 μL	90 μL
MgCl_2_ 100 mM	0.1 μL	75 μL
GTP 100 mM	0.04 μL	30 μL
DTT 100 mM	0.32 μL	240 μL
Nuclease-free water	0.79 μL	592.5 μL

***Note:*** When diluting the stock triton X-100 solution to 10%, warm up both the triton X-100 and the nuclease-free water at 56°C, for the triton X-100 to dissolve properly.

10. Validate gDNA or cDNA pre-amplification genotyping primers using bulk gDNA or cDNA (usually extracted from cell lines):
  a. Set up the following reaction for each primer pair:

**Table:** Pre-amplification genotyping primer testing reaction (bulk gDNA/cDNA)

Reagent	1 reaction
2× KAPA HiFi HotStart ReadyMix	5 μL
2× mock RT buffer	2 μL
Template gDNA (∼5 ng/μL) or cDNA (∼300 ng/μL)	1 μL
Forward pre-amplification primer 4 μM	1 μL
Reverse pre-amplification primer 4 μM	1 μL
  b. Set up a no template negative control reaction and a positive control reaction with validated primers (examples in Table S1).
  c. Run the following PCR program:

**Table:** PCR cycling conditions

Steps	Temperature	Time	Cycles
Initial Denaturation	98°C	3 min	1
Denaturation	98°C	20 sec	40 cycles
Annealing	67°C	30 sec	
Extension	72°C	6 min	
Final extension	72°C	5 min	1
Hold	12°C	Hold
  d. Run the PCR product on a 2% agarose gel and select the pair with the strongest and most specific amplification. Examples are shown in Figure 4.
    **CRITICAL:** If some genotyping primers do not amplify properly, do not adjust the above buffers or PCR conditions, but design new primer pairs instead (these conditions simulate the protocol).

**Figure 4:**
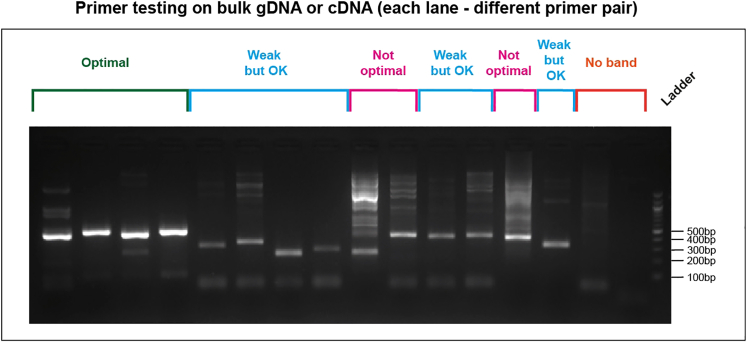
Testing genotyping primers in a bulk reaction 2% agarose gel electrophoresis where each lane shows bulk amplification using a distinct primer pair. The pairs indicated in green or blue can be tested in single cells. The pairs indicated in pink or orange should instead be re-designed. gDNA and cDNA extracted form cell lines can be used to test gDNA and cDNA primers, respectively.

### Preparation 3: Design and validate target-specific nested genotyping primers

**Timing: 2 days**

Target-specific nested genotyping primers used in PCR1 need to be designed and tested in a similar way as pre-amplification primers (preparation 2). If using both gDNA and cDNA pre-amplification primers, design both gDNA and cDNA nested primers. If using only gDNA pre-amplification primers, design only gDNA nested primers. If using a single pre-amplification primer pair to target both gDNA and cDNA, design a single nested primer pair.

11. Design gDNA nested genotyping primer pairs for each locus using Primer3, Primer Blast, or another tool for primer design. Design at least 2 pairs per locus.
  a. Use the following criteria:
    i. Primers should be nested within the gDNA pre-amplification primers designed in preparation 2, point 7. If not possible, these primers can be the same as the ones designed in preparation 2, point 7, but this reduces the amplification specificity.
    ii. Aim for an amplicon size 150–500 bp, if possible.
    iii. The 5′ end of either the forward or the reverse primer must be within 140 bp of the mutation. This is essential for the mutation to be covered during sequencing with 150 bp reads.
    iv. Aim for a primer length 19–25 bp.
    v. Melting temperature should be 57°C–63°C; GC content should be 20%–80%.
    vi. Check for primer specificity against a genomic and transcriptomic reference, using Primer Blast. If possible, select pairs with no/minimal non-specific binding.
    vii. If possible, design the primers to anneal to the introns adjacent to the mutant exon, which confers specificity for gDNA over cDNA. The primers can also anneal to intron-exon junctions. This may not be possible if the intronic region is far away from the mutation.
  b. Order the primers and reconstitute them at 100 μM in TE buffer.
  c. Prepare 5 μM primer pair dilutions in nuclease-free water to be used in preparation 3, point 13, by mixing 90 μL of nuclease-free water with 5 μL of forward primer and 5 μL of reverse primer.
12. If cDNA pre-amplification primers are used, design cDNA nested genotyping primer pairs for each locus using Primer3, Primer Blast, or another tool for primer design. Design at least 2 pairs per locus.
  a. Use the following criteria:
    i. Same as criteria 11ai–11avi for gDNA primers, checking for specificity only against a transcriptomic reference, and making sure the primers are nested to the cDNA pre-amplification primers designed in preparation 2, point 8.
    ii. If possible, design the primers to anneal to the exons adjacent to the mutant exon, which confers specificity for cDNA over gDNA. This is required if you want to use both gDNA and cDNA genotyping primers. The primers can also anneal to the junction between two exons.
  b. Order the primers and reconstitute them at 100 μM in TE buffer.
  c. Prepare 5 μM primer pair dilutions in nuclease-free water to be used in preparation 3, point 13, by mixing 90 μL of nuclease-free water with 5 μL of forward primer and 5 μL of reverse primer.
13. Validate gDNA or cDNA nested genotyping primers using bulk gDNA or cDNA (usually extracted from cell lines):
  a. Set up the following reaction for each primer pair:

**Table:** Nested genotyping primer testing reaction (bulk gDNA/cDNA)

Reagent	1 reaction
KAPA 2G Ready Mix	3.125 μL
5 μM dilution of nested primer pair	0.375 μL
Template gDNA (∼5 ng/μL) or cDNA (∼300 ng/μL)	1.5 μL
Nuclease-free water	1.25 μL
  b. Set up a no template negative control reaction and a positive control reaction with validated nested primers (examples in Table S1).
  c. Run the following PCR program:

**Table:** PCR cycling conditions

Steps	Temperature	Time	Cycles
Initial Denaturation	95°C	3 min	1
Denaturation	95°C	15 sec	35 cycles
Annealing	60°C	20 sec	
Extension	72°C	1 min	
Final extension	72°C	5 min	1
Hold	12°C	Hold
14. Run the PCR product on a 2% agarose gel and select the pair with the strongest and most specific amplification. Examples are shown in Figure 4.

***Note:*** We recommend validating both pre-amplification and nested genotyping primers simultaneously.

***Note:*** If a nested primer pair does not work efficiently, you can start combining one nested and one pre-amplification primer to check whether some of these pairs will be successful. Remember that the start of at least one primer needs to be within 140 bp of the mutation.

### Preparation 4: Validate target-specific genotyping primers in single cells

**Timing: 1–10 days**

**Figure 5:**
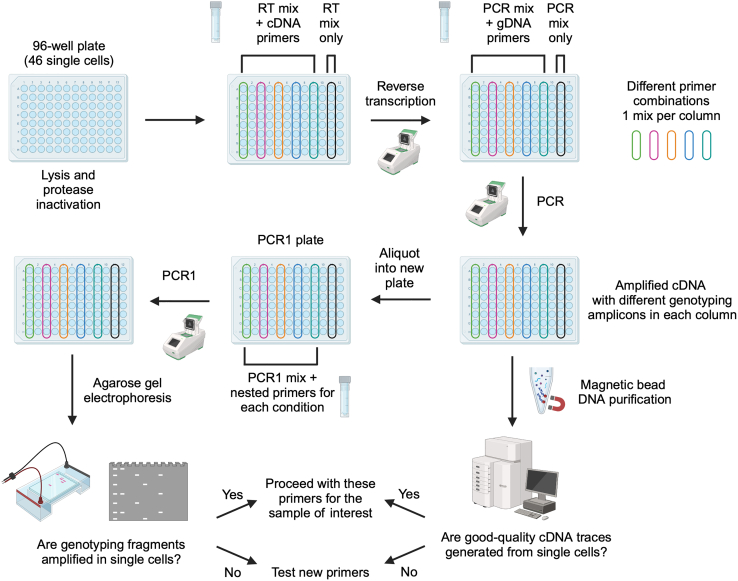
Genotyping primer testing in single cells After lysis and protease inactivation, RT and pre-amplification PCR are performed with different genotyping primer mixes that need to be tested. Each combination of pre-amplification genotyping primers should be tested in 8 single cells (one column of a 96-well plate). The last column is used as a positive control, where RT-PCR is performed without addition of targeted genotyping primers. Following RT-PCR, a portion of the material is purified with AMPure XP magnetic beads, keeping single cells separate, to evaluate whether cDNA libraries are efficiently generated from single cells (using a Fragment Analyzer or Bioanalyzer). Another portion of the remaining material from the original plate is transferred to a new 96-well plate, again keeping single cells separate, and used as the template for genotyping PCR1 reactions with nested genotyping primers. The final PCR product is run on a 2% agarose gel to evaluate whether genotyping amplicons are generated consistently in single cells.

Once successful pre-amplification and nested primers are identified in preparation 2 and 3, these primers need to be validated in single cells, in a simulation of the TARGET-seq+ protocol. These pilot experiments will define whether (a) genotyping amplicons are generated from single cells; and (b) whether single-cell cDNA libraries are successfully generated in presence of genotyping primers (Figure 5). We suggest testing each primer combination on at least 8 single cells (1 column of a 96-well plate).

***Note:*** For preparation 4, it is not necessary to use samples with specific mutations, as only the efficiency of amplicon generation, as opposed to mutation detection, is being evaluated here.

***Note:***Preparation 4, point 15 (prior to PCR) should be performed in a designated pre-PCR clean area, ideally in a biosafety cabinet.

15. Work with one plate of sorted single cells prepared in preparation 1, point 3. Keep the plate on dry ice until ready for protease inactivation. Perform protease inactivation and RT-PCR pre-amplification steps as described in part 3, with the following differences:
  a. Double all the volumes. After PCR, each well should contain ∼20 μL of material.
  b. Pipette by hand, because handling multiple mastermixes is not practical on liquid handling platforms.
  c. When preparing the RT mix, prepare as many RT mixes as there are cDNA primer conditions to test. For example, if you are testing 3 different primer combinations (for 3 different patient samples, for example), prepare 3 RT mixes containing these different primer combinations. Also, prepare one positive control RT mix without genotyping primers. Dispense each RT mix into a single column of the 96-well plate (Figure 5).
  d. The considerations from point 15c apply to the PCR mix as well (Figure 5).

**Pause point:** After PCR, the plate can be stored at −20°C for 6 months.

16. Once the RT-PCR is complete, dilute and purify the cDNA:
  a. Dilute the volume in each well 1:2 with nuclease-free water.
  b. Purify half of the volume (20 μL) from each well with AMPure XP beads, as described in preparation 1, point 5.
  c. Freeze the RT-PCR plate containing diluted cDNA-amplicon mix which will be used in preparation 4, point 18 (can be stored at −20°C for 6 months).
17. Check cDNA quality from each well by capillary electrophoresis, such as using a Bioanalyzer (Agilent), Fragment Analyzer (Agilent) or TapeStation (Agilent).
  a. If single-cell cDNA libraries are as shown in Figure 6A, the genotyping primers did not interfere with cDNA amplification.

**Figure 6:**
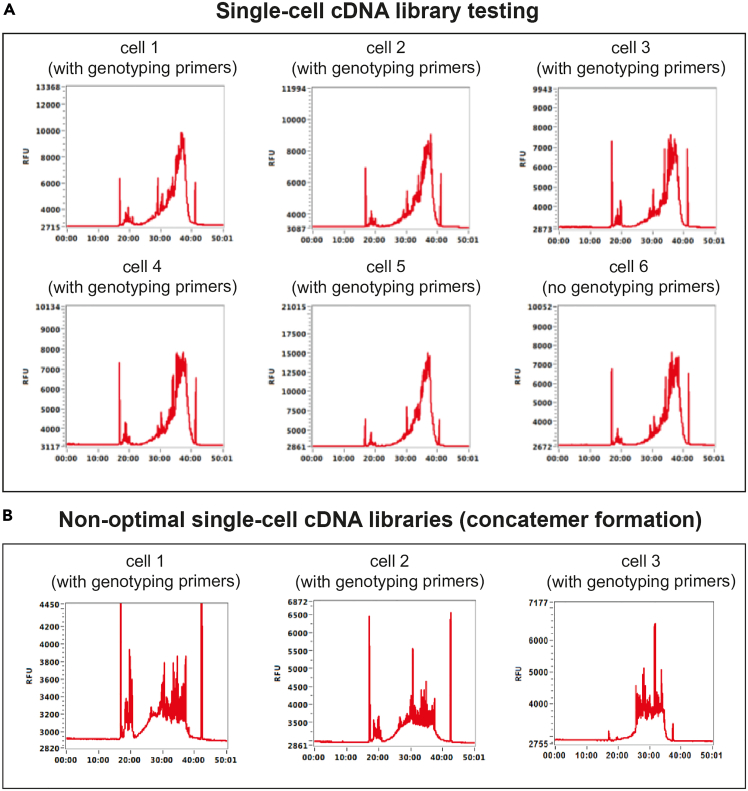
Optimal and non-optimal single-cell cDNA libraries (A) Optimal single-cell cDNA libraries generated via TARGET-seq+. Cell 1 – 5 indicate single-cell traces generated in presence of genotyping primers. Cell 6 indicates a cDNA library generated in absence of genotyping primers (positive control). (B) Non-optimal single-cell cDNA libraries generated via TARGET-seq+ in presence of genotyping primers showing formation of primer concatemers.
  b. If single-cell cDNA libraries are as shown in Figure 6B, the genotyping primers interfered with cDNA amplification. If you used a single primer pair, re-design primers and re-test them in single cells. If multiple primer pairs were used in the same reaction, repeat preparation 4, point 15 (Figure 5), this time having a single primer pair per RT and PCR mix. This will help to identify the problematic primer pair to be re-designed and re-tested.
18. If cDNA is successfully amplified from single cells (Figure 6A), the final preparation step is to test whether all genotyping amplicons are successfully generated from single cells (Figure 5). To test single-cell genotyping efficiency:
  a. Make a genotyping PCR1 master mix for each pair of nested PCR1 genotyping primers:

**Table:** Single-cell genotyping PCR1 testing reaction

Reagent	1 reaction
KAPA 2G Robust HS Ready Mix	3.125 μL
Nuclease-free water	1.25 μL
Nested genotyping forward/reverse primer pair 5 μM	0.375 μL
  b. Transfer a 1.5 μL aliquot from each well of the RT-PCR plate containing diluted cDNA-amplicon mix (preparation 4, point 16) into each genotyping PCR1 reaction. Set up a minimum of 6 reactions per condition. Set up a positive control with bulk gDNA or cDNA and a negative control with the no-template control (Figure 7A).

**Figure 7:**
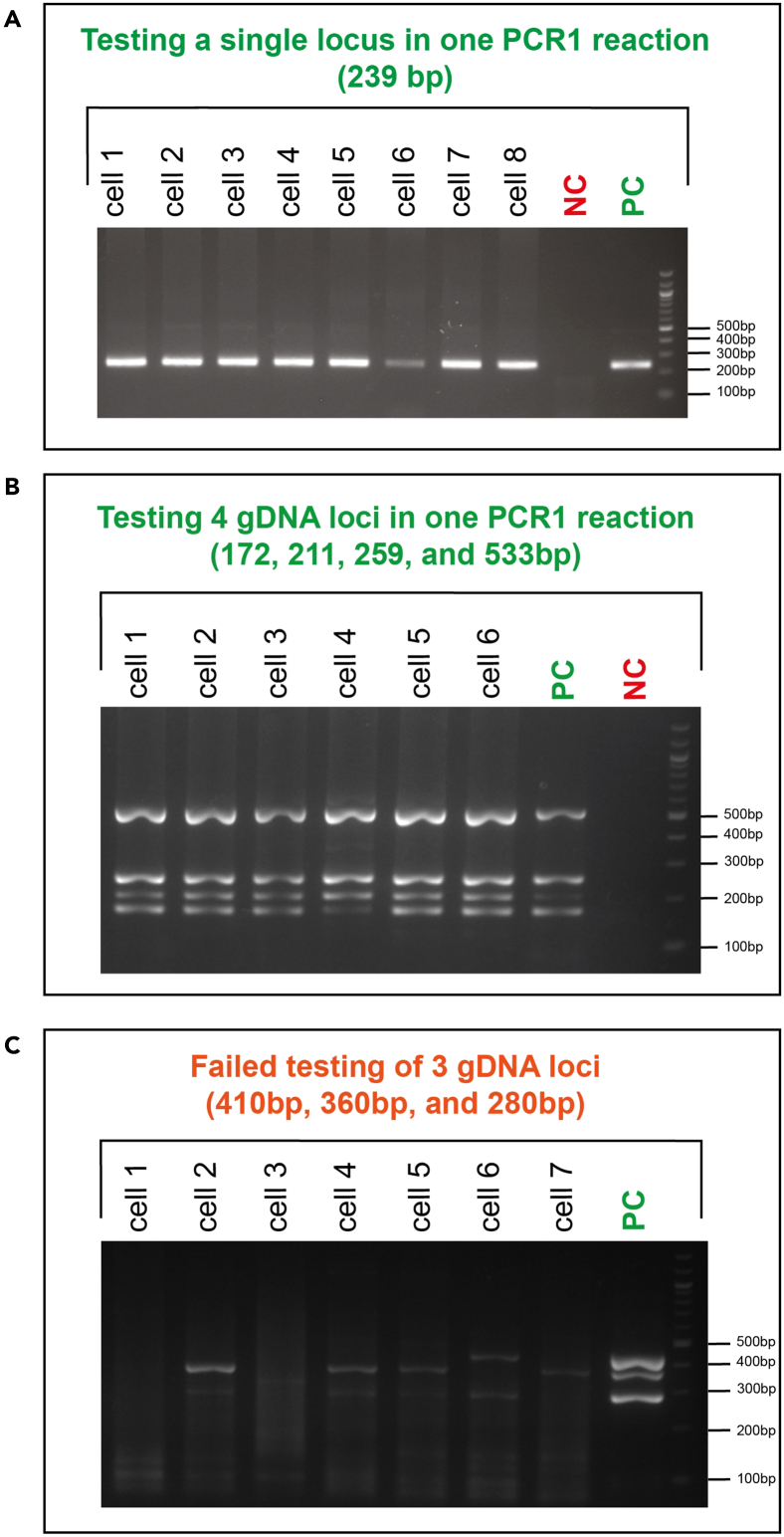
Optimal and non-optimal genotyping amplicons in single cells (A) 2% agarose gel electrophoresis showing optimal amplification of a single 239 bp gDNA genotyping amplicon in single cells. (B) 2% agarose gel electrophoresis showing optimal multiplexed amplification of 4 genotyping amplicons in single cells. Expected amplicon sizes shown above. In this example, 4 loci were amplified from gDNA. Note that amplification from cDNA is expected to be less consistent than from gDNA. (C) As in B but showing non-optimal amplification of 3 genotyping amplicons in single cells. NC, negative control (no-template); PC, positive control (bulk gDNA or cDNA).
  c. Repeat this for each nested genotyping PCR1 primer pair in a separate set of reactions.
  d. Additionally, to validate whether multiple loci are efficiently amplified in a single PCR1 reaction, set up multiplexed reactions that combine multiple nested PCR1 primer pairs in a single reaction (Figure 7B).
    ***Note:*** Due to the lower efficiency of cDNA amplification, gDNA and cDNA amplicons should usually be kept in separate PCR1 reactions. Moreover, we advise limiting the number of amplicons per PCR1 reaction to 4 (details in part 6). Hence, if > 4 amplicons are present, combine them in separate PCR1 reactions.
  e. Run the PCR program from preparation 3, point 13c.
19. Run each PCR product on a 2% agarose gel to check how many cells display successful amplification. Troubleshooting 13.
  a. If primers show acceptable single-cell amplification (ideally at least 5/6 strong and specific bands per locus; Figures 7A and 7B) and if single-cell cDNA library generation was successful (Figure 6A), these primers can be used on the samples of interest.
  b. If primers show very weak or no amplification (Figure 7C), these primers need to be re-designed. In our experience, the problem is usually due to non-specific pre-amplification genotyping primers. Re-check your bulk testing results (preparation 2 and 3) to identify which primer pair may have shown weak amplification in bulk.
  c. Follow this workflow to find suitable primers for all loci.

### Preparation 5: Preparation of barcoded primers for the main experiment

Once all pre-amplification and nested genotyping primers are validated, you are ready for the execution of TARGET-seq+ on samples of interest. At this stage, order and prepare all primers necessary for the protocol (sequences listed in the key resources table and Table S2).

20. Order the 384 oligo(dT)-ISPCR barcodes^3^ (Table S2) for the barcoded lysis buffer (part 1) in plate format, reconstituted at 100 μM in TE buffer. Keep the 384 barcoded oligo(dT)-ISPCR barcodes at −80°C prior to use.
21. Order HPLC-purified target-specific pre-amplification genotyping primers validated in preparation 2 and 4 (required for part 3). Reconstitute primers at 100 μM in RNase-free TE buffer. Keep reconstituted pre-amplification genotyping primers at −20°C prior to use.
22. Order the Access Array Barcode Library for Illumina Sequencers-384, Single Direction (four 96-well plates containing 40 μL of 2 μM primer pair per well; 384 barcoded primer pairs in total), required for part 6. We refer to these as “barcoded genotyping PCR2 primers”.
  a. Prepare two fresh 384-well plates.
  b. Transfer 20 μL of 2 μM barcoded genotyping PCR2 primers into each of the two fresh 384-well plates, following these steps:
    i. Aliquot each of the four original 96-well plates into a separate quadrant of the fresh 384-well plate (A1 into quadrant 1, A2 into quadrant 2, etc.).
    ii. Hereafter, we refer to these 384-well plates as “Access Array 2 μM stock plates”.
    iii. Keep the Access Array 2 μM stock plates at −20°C prior to use.

***Note:*** Order sufficient Access Array Barcode Library for Illumina Sequencers-384, Single Direction kits, for the approximate number of plates you plan to sort, considering that each genotyping PCR2 plate requires 1.2 μL of primer pair/well.

23. Order the CS1, LCS1, CS2, CS2rc, P5-seq, and i5-seq custom sequencing primers required for genotyping and cDNA library sequencing. Sequences are listed in the key resources table.
  a. Reconstitute the primers at 100 μM in RNase-free TE buffer.
  b. Prepare single-use aliquots for each primer:
    i. Primers and volumes required depend on the Illumina sequencing platform you will use to sequence genotyping libraries.
  c. Keep reconstituted single-use aliquots at −20°C prior to use.

**CRITICAL:** Handle all the above primers in a designated pre-PCR clean area, ideally in a biosafety cabinet, to avoid contamination. Some sequencing primers contain locked nucleic acid (LNA) modifications and should always be aliquoted and stored as single-use aliquots at −80°C.

## Key resources table

**Table:** 

REAGENT or RESOURCE	SOURCE	IDENTIFIER
**Antibodies**		

BV421 anti-human CD38 (clone HIT2) (1:20 dilution)	BioLegend	Cat# 303526; RRID:AB_10983072
BV605 anti-human CD10 (clone HI10a) (1:40 dilution)	BioLegend	Cat# 312222; RRID:AB_2562157
BV785 anti-human CD117 (clone 104D2) (1:40 dilution)	BioLegend	Cat# 313238; RRID:AB_2629837
BB515 anti-human CD45RA (clone HI100) (1:40 dilution)	BD	Cat# 564552; RRID:AB_2738841
PE anti-human CD123 (clone 6H6) (1:40 dilution)	BioLegend	Cat# 306006; RRID:AB_314580
PE/Dazzle 594 anti-human CD49f (clone GoH3) (1:160 dilution)	BioLegend	Cat# 313626; RRID:AB_2616782
PE/Cy7 anti-human CD90 (clone 5E10) (1:20 dilution)	BioLegend	Cat# 328124; RRID:AB_2561693
APC anti-human CD34 (clone 581) (1:160 dilution)	BioLegend	Cat# 343510; RRID:AB_1877153
PE/Cy5 anti-human CD2 (clone RPA-2.10) (1:160 dilution)	BioLegend	Cat# 300210; RRID:AB_314034
PE/Cy5 anti-human CD3 (clone HIT3a) (1:320 dilution)	BioLegend	Cat# 300310; RRID:AB_314046
PE/Cy5 anti-human CD4 (clone RPA-T4) (1:160 dilution)	BioLegend	Cat# 300510; RRID:AB_314078
PE/Cy5 anti-human CD8a (clone RPA-T8) (1:320 dilution)	BioLegend	Cat# 301010; RRID:AB_314128
PE/Cy5 anti-human CD11b (clone ICRF44) (1:160 dilution)	BioLegend	Cat# 301308; RRID:AB_314159
PE/Cy5 anti-human CD14 (clone 61D3) (1:160 dilution)	eBioscience	Cat# 15-0149-42; RRID:AB_2573058
PE/Cy5 anti-human CD19 (clone HIB19) (1:160 dilution)	BioLegend	Cat# 302210; RRID:AB_314240
PE/Cy5 anti-human CD20 (clone 2H7) (1:160 dilution)	BioLegend	Cat# 302308; RRID:AB_314256
PE/Cy5 anti-human CD56 (clone MEM188) (1:80 dilution)	BioLegend	Cat# 304608; RRID:AB_314450
PE/Cy5 anti-human CD235ab (clone HIR2) (1:320 dilution)	BioLegend	Cat# 306606; RRID:AB_314623

**Chemicals, peptides, and recombinant proteins**		

IMDM	Gibco	Cat# 21056023
DPBS	Thermo Fisher Scientific	Cat# 14190169
Fetal bovine serum	Sigma-Aldrich	Cat# F7524
DNase I	Roche	Cat# 11284932001
Ficoll-Paque PLUS	GE Healthcare	Cat# 17-1440-03
7-AAD	BioLegend	Cat# 420404
SDS 10%	Sigma-Aldrich	Cat# L4509
Triton X-100	Sigma-Aldrich	Cat# T8787
dNTPs (10 mM each)	Thermo Scientific	Cat# R0193
Poly-ethylene glycol 8000 (40% solution)	Sigma-Aldrich	Cat# P1458
Recombinant RNase inhibitor (40 U/μL)	Takara	Cat# 2313B
Protease	QIAGEN	Cat# 19155
Nuclease-free water	Invitrogen	Cat# AM9937
Tris-HCl 1 M pH 8.0	Thermo Scientific	Cat# 15893661
NaCl 5 M	Invitrogen	Cat# AM9760G
MgCl2 1 M	Invitrogen	Cat# AM9530G
GTP solution, Tris buffered	Thermo Scientific	Cat# R1461
Dithiothreitol (DTT), 0.1 M solution	Thermo Scientific	Cat# 707265ML
UltraPure agarose	Invitrogen	Cat# 16500-500
Ethidium bromide solution	Invitrogen	Cat# 15585-011

**Critical commercial assays**		

CompBeads	BD Biosciences	Cat# 552843
ERCC RNA Spike-In Mix	Invitrogen	Cat# 4456740
Maxima H Minus Reverse Transcriptase (200 U/μL)	Thermo Scientific	Cat# EP0753
KAPA HiFi HotStart ReadyMix	Roche	Cat# 07958935001
KAPA 2G Robust HS Ready Mix	Sigma-Aldrich	Cat# KK5702
FastStart High Fidelity PCR System, dNTPack	Sigma-Aldrich	Cat# 4738292001
Nextera XT DNA Library Preparation Kit (96 samples)	Illumina	Cat# FC-131-1096
Nextera XT Index Kit Set v2 Set A	Illumina	Cat# FC-131-2001
Nextera XT Index Kit Set v2 Set C	Illumina	Cat# FC-131-2003
Access Array Barcode Library for Illumina Sequencers-384, Single Direction	Fluidigm	Cat# 100-4876
AMPure XP Beads	Beckman Coulter	Cat# A63881
Qubit dsDNA HS Assay Kit	Invitrogen	Cat# Q32854
High Sensitivity NGS Fragment Analysis Kit (1–6,000 bp)	Agilent	Cat# DNF-474-0500
Agilent High Sensitivity DNA Kit	Agilent	Cat# 5067-4626
Agilent Tapestation HS D1000 ScreenTape	Agilent	Cat# 5067-5583
Agilent Tapestation HS D1000 Reagents	Agilent	Cat# 5067-5584

**Deposited data**		

Targeted DNA sequencing, raw data	Jakobsen et al.^1^	EGA: EGAS00001007358
TARGET-seq+ single-cell RNA sequencing, raw data	Jakobsen et al.^1^	EGA: EGAS00001007358
TARGET-seq+ single-cell genotyping, raw data	Jakobsen et al.^1^	EGA: EGAS00001007358
TARGET-seq+ single-cell RNA sequencing, processed raw counts	Jakobsen et al.^1^	Figshare: https://doi.org/10.25446/oxford.23576379
TARGET-seq+ single-cell genotyping data, processed allelic counts	Jakobsen et al.^1^	Figshare: https://doi.org/10.25446/oxford.23576421
TARGET-seq+ single-cell metadata and genotypes	Jakobsen et al.^1^	Figshare: https://doi.org/10.25446/oxford.23576262

**Oligonucleotides**		

Oligo(dT)-ISPCR (HPLC purification): AAGCAGTGGTATCAACGCA GAGTACTTTTTTTTTTTTTT TTTTTTTTTTTTTTTTVN	Picelli et al.^4^	N/A
Barcoded oligo(dT)-ISPCR primers	Biomers (design: Rodriguez-Meira et al.^3^)	N/A
TSO-LNA (RNase-free HPLC purification): /5Biosg/AAGCAGTGGTATCAACGCAGAGTACATrGrG+G	IDT (design: Picelli et al.^4^)	N/A
ISPCR primer (HPLC purification): AAGCAGTGGTATCAACGCAGAGT	IDT (design: Picelli et al.^4^)	N/A
See Table S1 for validated target-specific genotyping primers used in the pre-amplification step (HPLC purification)	IDT (design: Jakobsen et al.^1^ and Turkalj et al.^10^)	N/A
See Table S1 for validated target-specific nested barcoded genotyping primers used in the PCR1 barcoding step (standard desalting)	IDT (design: Jakobsen et al.^1^ and Turkalj et al.^10^)	N/A
See Table S2 for custom transcriptome i5 index primers (HPLC purification)	IDT (design: Jakobsen et al.^1^)	N/A
P5-SEQ primer (PAGE purification): GCCTGTCCGCGGAAGCAGTGGT ATCAACGCAGAGTTGC∗T	Rodriguez-Meira et al.^3^	N/A
I5-SEQ primer (PAGE purification): AGCAACTCTGCGTTGATACCACT GCTTCCGCGGACAGG∗C	IDT (design: Jakobsen et al.^1^)	N/A
LCS1 sequencing primer (HPLC purified): GGCGACCACCGAGATCTACACTGACG ACATGGTTCTACA	IDT	N/A
CS2 sequencing primer (HPLC purified): T+AC+GGT+AGCAGAGACTTGGTCT	IDT	N/A
CS2rc sequencing primer (HPLC purified): A+GAC+CA+AGTCTCTGCTACCGTA	IDT	N/A

**Software and algorithms**		

Bcl2fastq (v.2.20)	Illumina	https://support.illumina.com/sequencing/sequencing_software/bcl2fastq-conversion-software.html
Python (v.3)	Python Software Foundation	https://www.python.org
CGAT-core	Bioconda	https://github.com/cgat-developers/cgat-core
Samtools	HTSlib	http://www.htslib.org/download/
Cutadapt (v.3.4)	Bioconda	https://cutadapt.readthedocs.io/en/stable/
FastQC (v.0.11.9)	Bioconda	https://www.bioinformatics.babraham.ac.uk/projects/fastqc/
MultiQC (v.1.11)	Bioconda	https://multiqc.info
STAR (v.2.7.10a)	GitHub; Dobin et al.^13^	https://github.com/alexdobin/STAR
R (v.4.2.1)	R-Project	https://www.r-project.org
ggplot2 (v.3.3.6)	CRAN	https://ggplot2.tidyverse.org
Primer3Plus	Untergasser et al.^11^	https://www.primer3plus.com
Primer-BLAST	National Library of Medicine; Ye et al.^12^	https://www.ncbi.nlm.nih.gov/tools/primer-blast/
TARGET-seq genotyping pipeline	GitHub; Rodriguez-Meira et al.^3^	https://github.com/albarmeira/TARGET-seq
infSCITE	GitHub; Kuipers et al.^14^	https://github.com/cbg-ethz/infSCITE
Custom code for TARGET-seq+ analysis	Jakobsen et al.^1^	https://github.com/asgerjakobsen/TARGET-seq-plus https://github.com/asgerjakobsen/TARGET-seq-plus-RNA

**Other**		

12.5 mL GRIPTIP, sterile, filter	INTEGRA Biosciences	Cat# 6455
High Volume MANTIS chip	FORMULATRIX	Cat# MCHVSMR6
Agencourt AMPure XP beads (or equivalent)	Beckman Coulter	Cat# A63881
Framestar PCR plate 384 well, skirted	Azenta Life Sciences	Cat# 4ti-0384/C
Framestar 96-well semi-skirted PCR plate	Azenta Life Sciences	Cat# 4ti-0900/C
MicroAmp Optical 96-well reaction plate	Thermo Fisher Scientific	Cat# N8010560
Axygen 96-well clear V-bottom 500 mL polypropylene deep-well plate	Corning	Cat# P-96-450V-C
Adhesive PCR Plate Seals (plastic)	Thermo Fisher Scientific	Cat# AB0558
Self-adhesive Plate Seal, aluminum, thick 60 mm	STARLAB (UK) Ltd.	Cat# E2796-0792
DNA LoBind tube 1.5 mL	Eppendorf	Cat# 022431021
NucleoCounter NC-3000 (or equivalent)	ChemoMetec	Cat# 991-3001
NC-Slide A8 (or equivalent)	ChemoMetec	Cat# 942-0003
Solution 13 (or equivalent)	ChemoMetec	Cat# 910-3013
CoolRack XT PCR384 thermoconductive tube rack for 384-well PCR plates (or equivalent)	Azenta Life Sciences	Cat# BCS-538
Centrifuge 5430 R (or equivalent)	Eppendorf	Cat# 5428000655
Centrifuge 5910 R (or equivalent)	Eppendorf	Cat# 5943000061
MPS 1000 Mini plate spinner (or equivalent)	Labnet	Cat# C1000
ProFlex 96-well PCR system (or equivalent)	Thermo Fisher Scientific	Cat# 4484075
ProFlex 384-well PCR system (or equivalent)	Thermo Fisher Scientific	Cat# 4484077
Invitrogen Qubit 3 Fluorometer (or equivalent)	Thermo Fisher Scientific	Cat# 15387293
Qubit assay tubes (or equivalent)	Thermo Fisher Scientific	Cat# Q32856
2100 Bioanalyzer Instrument	Agilent	Cat# G2939BA
4200 TapeStation System (or equivalent)	Agilent	Cat# G2991BA
Magnetic stand-96 (or equivalent)	Thermo Fisher Scientific	Cat# AM10027
Magnetic separation rack, 0.2 mL tubes (or equivalent)	EpiCypher	Cat# 10-0008
GelDoc Go Gel imaging system (or equivalent)	Bio-Rad	Cat# 12009077
PowerPac Basic power supply (or equivalent)	Bio-Rad	Cat# 1645050
Fisherbrand Midi Plus Horizontal Gel System (or equivalent)	Thermo Fisher Scientific	Cat# 11833293
MA900 Multi-application cell sorter (or equivalent)	Sony	N/A
MANTIS automated liquid dispenser (or equivalent)	Formulatrix	N/A
Mosquito HTS Nanolitre Liquid Handler (or equivalent)	SPT Labtech	N/A
VIAFLO 96/384 electronic pipette (or equivalent)	INTEGRA Biosciences	N/A

## Materials and equipment

***Note:*** FACS buffer and thawing media recipes listed below apply to thawing and staining procedures involving human cryopreserved bone marrow/peripheral blood mononuclear cells.

**Table:** FACS buffer

Reagent	Amount
Fetal Bovine Serum (FBS)	50 mL
DNase I (10 mg/mL)	500 μL
IMDM (no phenol red)	450 mL
**Total**	**500.5 mL**

Store at 4°C for a maximum of two weeks.

**Table:** Thawing media for cryopreserved human bone marrow/peripheral blood mononuclear cells

Reagent	Amount
Fetal Bovine Serum (FBS)	5 mL
DNase I (10 mg/mL)	500 μL
FACS buffer	45 mL
**Total**	**50.5 mL**

Make fresh on the day of the sort.

***Note:*** Pass the FACS buffer and thawing media through a sterile filter prior to storage.

**Template-switching oligo (TSO):** Reconstitute the TSO to 100 μM in RNase-free TE buffer and prepare single-use aliquots (∼72 μL for 4 plates with 192 cells). Keep the TSO cold. Work in a pre-PCR area, in a biosafety cabinet. Snap freeze the TSO aliquots and store at −80°C prior to use.

**CRITICAL:** Do not freeze-thaw the TSO. Do not use TSO older than 6 months.

**ERCC stock:** Serially dilute the ERCC stock (1×) to 1:400,000 in RNase-free TE buffer and prepare single-use aliquots (∼30 μL for 8 plates with 192 cells). Keep ERCC cold. Work in a pre-PCR area, in a biosafety cabinet. Snap freeze the ERCC aliquots and store at −80°C prior to use.

**Triton X-100 stock:** Prepare aliquots of Triton X-100 10%. First pre-warm both the Triton X-100 and nuclease-free water at 56°C for 10 min. Mix 100 μL of pre-warmed Triton X-100 with 900 μL of pre-warmed nuclease-free water.

**10 μM barcoded oligo(dT)-ISPCR stock plates:** Transfer 3 μL of barcoded oligo(dT)-ISPCR from the 100 μM plate (preparation 5) into 27 μL of RNase-free TE buffer. Use a multichannel system to transfer the oligo(dT)-ISPCR between plates. Work in a pre-PCR area, in a biosafety cabinet. Snap freeze both plates and store at −80°C prior to use.

**CRITICAL:** Take great care not to cross-contaminate oligo(dT)-ISPCR barcodes from different wells.

## Step-by-step method details

### Part 1: Barcoded lysis buffer preparation

**Timing: 3–5 h**

**Figure 8:**
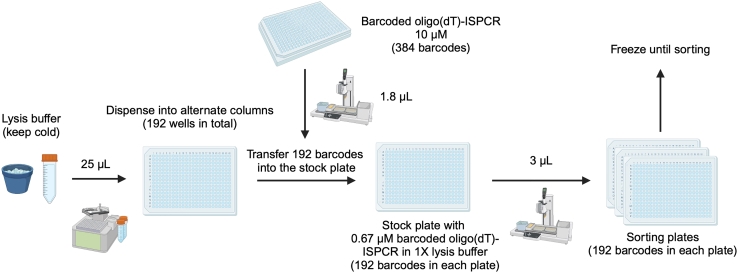
Barcoded lysis buffer preparation All the steps should be carried out in a biosafety cabined in a specialized pre-PCR environment. Prepare the lysis buffer and keep cold. Then dispense 25 μL of lysis buffer into alternate columns (192 wells) of a lysis stock plate using the Mantis. Next, transfer 1.8 μL of barcoded oligo(dT)-ISPCR primers from a 10 μM stock plate into the lysis stock plate using the Integra VIAFLO to obtain 0.67 μM barcoded oligo(dT)-ISPCR primers in 1× lysis buffer. Each well of the lysis stock plate should contain a unique oligo(dT)-ISPCR barcode. Next, using the Integra VIAFLO in repeat dispense mode, transfer 3 μL from the lysis stock plate into fresh 384-well plates, which will be used for single-cell sorting. Snap freeze the sorting plates on dry ice. This procedure generates a total of 8 sorting plates; if more are required, this needs to be repeated.

This step describes how to prepare 384-well plates containing lysis buffer with barcoded oligo(dT)-ISPCR primers (Figure 8). Each well of the lysis buffer plate will contain a unique oligo(dT)-ISPCR barcode which will be used as a cell barcode when sequencing the transcriptome libraries.

***Note:*** Lysis buffer plates can be prepared in advance of single-cell sorting and stored at −80°C for up to 1 month. It is possible to use 384-well or 96-well plates. Here, we describe the procedure for 384-well plates. If 96-well plates are used instead, we recommend doubling all the volumes. If using 384-well plates, consider leaving alternate columns of the plate empty (i.e. filling only 192 wells on each plate) as described below. This is because, during the FACS sort (part 2), the time taken to sort single cells into each plate should be kept below 20 min. When the target cell population is rare within the sample, it may be necessary to limit the sort to 192 wells per plate, as filling 384 wells may not be possible within this timeframe.

**CRITICAL:** The preparation of lysis buffer plates is critical for successful cDNA generation, and should be performed in a pre-PCR clean area, ideally in a biosafety cabinet, to avoid contamination from PCR products and RNases. Clean the biosafety cabinet and pipets with a cleaning agent to remove RNases (e.g., RNaseZap) and use RNase-free filter tips during the entire protocol. Moreover, all steps should be carried out on ice or a cool block.

***Note:*** The amount of ERCC to add may vary depending on tissue type. Please refer to Rodriguez-Meira et al., 2020,^3^ for additional details.

***Note:*** For preparing lysis plates and throughout many steps of the protocol, we use the MANTIS Microfluidic Liquid Dispenser (FORMULATRIX) and the INTEGRA VIAFLO 96/384 electronic pipettor. Note that the use of these platforms is not essential but increases throughput and reduces inconsistencies. For detailed pictures, video guidance, and instructions relative to the usage of these platforms, please refer to our prior GTAC protocol.^9^ We advise familiarizing yourself with the working procedures in advance.

***Optional:*** Those steps that we perform with the INTEGRA VIAFLO electronic pipettor with a 384-well 12.5 μL pipetting head can be performed with alternative multichannel pipette systems. Those steps that we perform with the MANTIS Microfluidic Liquid Dispenser can be performed with alternative multi-well dispensers. This applies to the entire protocol.

1. Prepare lysis buffer mix as outlined in the table below and keep it on ice:

**Table:** Lysis buffer

Reagent	Final concentration in the lysis buffer	Volume per well (μL)	Volume for 1536 wells (8 plates) + 21.5% dead volume (μL)
Nuclease-Free Water		1.94	3620
Poly-ethylene Glycol 8000 (40% solution)	6.7%	0.5	933
Triton X-100 (10% solution)	0.1%	0.03	56
ERCC 1:4e5	-	0.015	28
RNase Inhibitor	0.5 U/μL	0.04	75
dNTPs (10 mM/each)	0.67 mM/each	0.2	373
Protease (1.09 AU/mL)	27 mAU/mL	0.075	140
**Total**		**2.80**	**5225**

***Note:*** Ensure that PEG is fully mixed into solution, by adding to water and pipetting up and down until the liquid is clear, before adding the remaining reagents.

2 Dispense 25 μL of lysis buffer into each well in alternate columns of a fresh 384-well plate, leaving the other columns empty.

***Note:*** We use the MANTIS Liquid Handling Platform (FORMULATRIX) with a High Volume (HV) chip to dispense lysis buffer into each well of the stock plate.

3 Thaw the stock plate containing 10 μM barcoded oligo(dT)-ISPCR primers and spin down at 500 × g for 10 s.
4 Transfer 1.8 μL of the 10 μM barcoded oligo(dT)-ISPCR primers to the lysis buffer plate, to obtain a stock plate of 0.67 μM barcoded oligo(dT)-ISPCR primers in 1× lysis buffer, using the INTEGRA VIAFLO electronic pipettor with a 384-well 12.5 μL pipetting head. Pipette up and down to mix.

**CRITICAL:** Take great care to avoid any cross-well contamination of barcodes at this point!

***Note:*** When using the VIAFLO to transfer or mix viscous solutions (as the lysis buffer), keep the Dispense Speed low, to avoid retention of the volume inside the tips.

**Figure 9:**
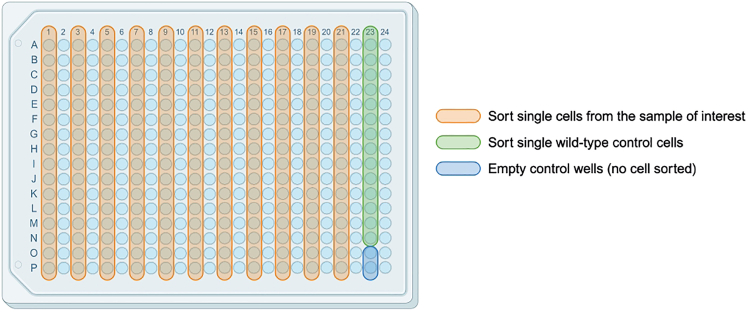
Plate sorting configuration The wild-type control cells should be sorted from a sample which is wild-type for all mutation loci being genotyped.

***Note:*** The volume in the lysis stock plate will be sufficient for 8 sorting plates containing 3 μL per well in alternate columns (192 wells per plate).

5. Cover the plate with a PCR film and spin down at 500 × g for 10 s.
6. Transfer 3 μL of lysis buffer containing barcoded oligo(dT)-ISPCR primers into each well in alternate columns of a sterile 384-well plate to obtain a “sorting plate” which single cells will be sorted into (Figure 9) using the INTEGRA VIAFLO electronic pipettor with a 384-well 12.5 μL pipetting head.

7. Centrifuge all the plates in a plate spinner at 500 × g for 10 s. Snap freeze the plates on dry ice. Store plates at −80°C until the sort.

**CRITICAL:** When freezing plates, always use aluminum instead of plastic adhesive seals.

**Pause point:** You can store the sorting plates containing barcoded 1× lysis buffer at −80°C for up to 1 month until the sort.

### Part 2: Sample preparation and single-cell sorting

**Timing: 1 day**

This step describes the protocol for sample processing and sorting single cells into plates containing lysis buffer prepared in part 1. We describe the conditions used for HSPCs from human bone marrow or peripheral blood. Other cell types may require variations of this protocol which will need to be optimized in advance.

***Note:*** Prepare FACS buffer, thawing media, and antibody dilutions the day before the sort.

***Note:*** Points 8–11 describe the procedures for thawing cryopreserved bone marrow or peripheral blood samples. If using fresh tissues, proceed to point 12. If working with other tissues, please follow relevant protocols for tissues dissociation and/or thawing to obtain single cell suspensions.

**CRITICAL:** To have appropriate single-cell genotyping controls and to control for batch effects in scRNA-seq, we sort cells from a wild-type control sample into each plate. Therefore, we always process a cryopreserved control sample in parallel with the sample(s) of interest. This control sample is also used for Fluorescence-Minus-One (FMO) controls and setting sorting gates. Take care not to cross-contaminate samples.

**CRITICAL:** Accurate single-cell sorting is critical for successful library generation. Use a cell sorter that can be accurately aligned to sort into 384-well plates. We use the Sony MA900 Multi-Application Cell Sorter, but other instruments can be used. If accurate alignment in 384-well plates is not possible with your instrument, consider performing this part of the protocol using 96-well plates instead.

8. Prepare for cryopreserved sample processing:
  a. Prepare a laminar flow cabinet for sterile work.
  b. Prepare a box of wet ice and place FACS buffer on ice.
  c. Bring cryopreserved samples from liquid nitrogen on dry ice.
  d. Set the water bath to 37°C and warm the thawing media and FBS.
9. Thaw the samples. Work with a maximum of two samples at a time:
  a. Place samples in the water bath and wait until they are 70–80% liquid. Proceed when there is still a small piece of frozen tissue inside the cryovial.
  b. Dropwise, add 1 mL of warm FBS to the sample using a P1000.
  c. Gently transfer all the volume into a 15 mL conical centrifuge Falcon tube.
  d. Dropwise, add 1 mL of warm thawing media to the tube. Hand-mix gently.
  e. Wash the cryovial with 1 mL of thawing media and add dropwise to the tube.
  f. Slowly, add 6 mL of thawing media to the tube to reach a total of 10 mL. After every 2 mL added, hand-mix gently.
10. When all samples are ready, centrifuge at 350 × g for 10 min at 21°C. Remove supernatant by pouring it gently and then blotting the tube on dry paper, to remove traces of liquid.
11. Resuspend cells in 1 mL of ice-cold FACS buffer and mix gently.
  a. Pass cells through a 35 μm cell strainer.
  b. Wash the 15 mL tube with 1 mL of FACS buffer. If starting cell number was > 20 million, add an additional 3 mL of FACS buffer, for a total of 5 mL.
12. Count cells either with trypan blue and a hemocytometer or using an automatic cell counter.
  a. Take note of total cell numbers and viability.
  b. Set aside an aliquot of approximately 500,000 cells for unstained, single stained, and FMO controls, and place them on ice.
13. Centrifuge the other samples at 350 × g for 5 min. Remove supernatant as in point 10. ∼50 μL of supernatant will remain in the tube.
14. Add Human Fc-blocking solution (1:20) and incubate at 4°C for 5 min.
15. Perform the antibody staining:
  a. Work without light.
  b. Add antibody mixes to each sample.
    ***Note:*** We add 50 μL of antibody mix if cell number is < 10 million or 100 μL of antibody mix for higher cell numbers. The antibody mixes and dilutions we use for staining primary human bone marrow HSPCs are in Table S3.
  c. Place samples at 4°C, in the dark, and incubate for 30–40 min.
    ***Note:*** If cells clump during this step and cannot be properly resuspended in the antibody mix, eliminate these with the pipette tip, given that they may lead to suboptimal staining.
16. Take the control cell aliquot set aside in point 12b and mix gently.
  a. Add cells to each 2× FMO mix and mix gently.
    ***Note:*** The 2× FMO mixes contain antibodies at twice the staining concentration; the concentration is brought to 1× by adding the cells.
  b. Place FMO tubes at 4°C, in the dark.
  c. Incubate for 30–40 min.
17. Wash all samples and FMO controls with 1 mL of ice-cold FACS buffer and centrifuge at 350 × g for 5 min at 4°C.
18. Remove supernatant and resuspend in ice-cold FACS buffer.
  a. Resuspend full stain samples in 500 μL–1 mL of FACS buffer.
  b. Resuspend FMOs in 100 μL of FACS buffer with 1:200 7-AAD, except the FMO corresponding to the 7-AAD channel, which should be resuspended in 100 μL of FACS buffer.
19. Set up the FACS panel on the cell sorter:
  a. Use single stains to set up voltages.
  b. Run each FMO control and perform “Manual Compensation” to optimize the compensation matrix.
  c. Set up the sorting gates.
20. Add 1:200 7-AAD viability dye to full stain samples and, if necessary, pass the cell suspension through a 35 μm cell strainer. Perform an enrichment sort for your target population using yield sorting mode.

***Note:*** The purpose of the enrichment sort is to obtain a sufficiently high proportion of target cells within the cell suspension, so that each plate can be filled within 20 min when sorting single cells. If the frequency of your target population is > 25%, this step is not necessary.

***Note:*** To minimize cell loss, pre-coat collection tubes with 1 mL of FACS buffer for 5 min.

21. Prepare the sorter for single-cell sorting. Load the 384-well plate holder.
22. Perform sorter calibration for 384-well plate sorting:
  a. Cover an empty 384-well plate (same model in which lysis buffer was aliquoted) with a plastic PCR adhesive seal and insert into the collection platform.
  b. Sort 30 droplets into each corner of the plate (positions A1, A24, P1, P24).
  c. Adjust the droplet position and repeat the process iteratively to center all the droplets in the middle of the wells.
  d. Remove the seal from the plate and sort 20 droplets into the same wells as in point 22b.
  e. If needed, adjust the positions iteratively until the droplets are deposited at the bottom of each well. Make sure there is no splashing on the sides of the wells.

**CRITICAL:** Improper calibration will result in failed library generation. Refer to our prior protocol^9^ for detailed graphical guidance on plate calibration.

23. Clean the surfaces around the sorter with RNase away.
24. Thaw plates containing lysis buffer with barcoded oligo(dT)-ISPCR primers from point 7 and centrifuge in a plate spinner at 500 × g for 20 s. Store at 4°C until sorting.
25. Proceed to single-cell sorting (Figure 9) of your target populations into the thawed plates containing lysis buffer with barcoded oligo(dT)-ISPCR primers from point 24.
  **CRITICAL:** Sort using a single-cell sorting purity mode and stringent doublet exclusion gates. We use a mode in which 50% of the droplet before and after the target droplet must be empty, and in which the target event needs to be within the 75% central part of the droplet.
  **CRITICAL:** Sort the cells at 21°C for a maximum of 20 min per plate. If your target population is rare and filling up a plate would take longer than 20 min, you should enrich your target population before sorting into plates to avoid RNA degradation (see point 20). Additionally, consider sorting only into alternate columns of every plate (i.e. leaving 192 wells/plate empty when aliquoting lysis buffer and sorting single cells) to reduce the time taken to sort into each plate.
  a. Carefully remove the seal from the lysis buffer plate and load onto the collection platform.
  b. On each plate, sort single cells from your sample of interest into 176 wells.
  c. When complete, remove the tube and flush the sample line. Load the wild-type control sample, and sort single cells from this sample into the remaining 14-15 wells. Leave 1-2 wells empty as no-template controls (Figure 9).
  d. Sort using “index sorting” mode to record the fluorescence intensity of each cell-surface marker which can be used for cell-surface proteomics analysis.
  e. Keep the event rate low (aim for < 10 events/s). Dilute the sample if needed.
26. Once finished, cover the plate carefully with an aluminum adhesive seal, centrifuge in a plate spinner at 500 × g for 20 s, and snap freeze on dry ice. Repeat points 25-26 for all plates.

**Pause point:** Sorted plates can be stored at −80°C for up to 3 months. Processing plates after 3 months is associated with higher rates of RNA degradation.

### Part 3: Lysis, reverse transcription, and pre-amplification

**Timing: 7 h**

**Figure 10:**
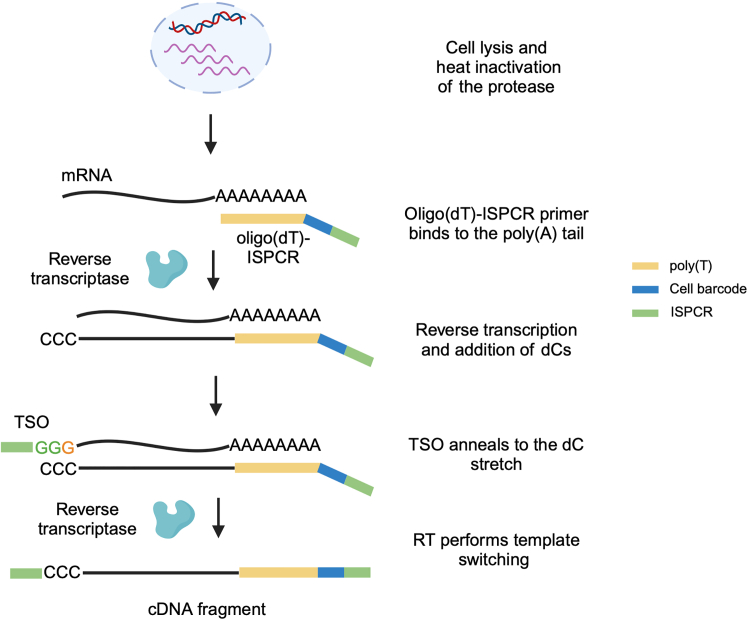
Schematic of the RT chemistry Sequence color-coding on the right. The cell is first lysed and subjected to heat inactivation of the protease. During the RT, the poly(dT) sequence of the barcoded oligo(dT) binds to the poly(A) tail of the mRNA. This primes the reverse transcriptase, which synthesizes the first cDNA strand. On reaching the 5′ end of the RNA template, the reverse transcriptase adds 2–5 untemplated dC nucleotides to the cDNA end. This enables the template switching reaction, where the TSO anneals to the dC nucleotides, the reverse transcriptase switches template strands and continues to replicate the TSO sequence to complete the first strand.

**Figure 11:**
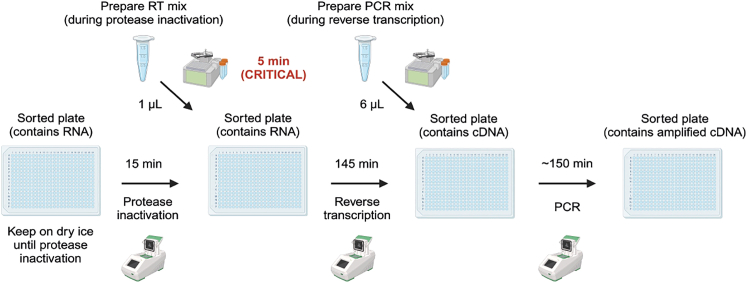
Execution of the RT-PCR steps All the steps should be carried out in a biosafety cabinet in a specialized pre-PCR environment. The sorted plate is transferred directly from dry ice onto a thermal cycler for protease inactivation. As soon as this step is finished, 1 μL of RT mix is added to each well using the Mantis. This is a critical step and needs to be performed within 5 min. As soon as the RT mix is added, the plate is placed onto a thermal cycler for the RT program to take place. Following RT, 6 μL of PCR mix (which contains targeted genotyping primers) are aliquoted into the plate using the Mantis. The plate is then subject to PCR outside of the pre-PCR environment. After PCR, the plate will contain a mix of amplified cDNA and genotyping amplicons.

**Figure 12:**
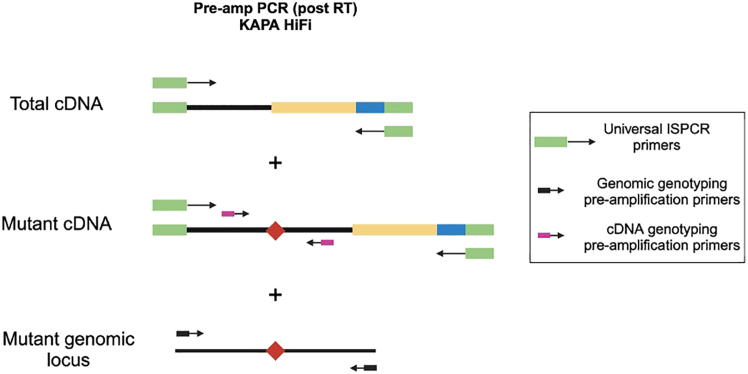
Schematic of simultaneous pre-amplification of cDNA and genotyping fragments in the PCR During PCR, full-length cDNA (for the single-cell transcriptome libraries) and genotyping loci (for single-cell genotyping libraries) are amplified in parallel. The full-length cDNA is amplified by a universal ISPCR primer which anneals to the ISPCR adapter sequences incorporated by the oligo(dT) and TSO during RT. Genotyping loci are amplified from gDNA and, optionally, from cDNA. gDNA targeted amplification is achieved by addition of targeted genomic genotyping primers which anneal to introns flanking the mutant exon. Moreover, genotyping cDNA primers may be used, which further enrich for the mutant locus from the cDNA, which is simultaneously amplified by ISPCR primers.

This section describes the procedures for cell lysis, protease heat inactivation, RT (Figures 10 and 11), and PCR pre-amplification of cDNA and targeted genotyping amplicons (Figure 12). At the end of this part, each well will contain pre-amplified full-length cDNA with single-cell 3′ oligo(dT) barcodes, along with targeted genotyping amplicons. The targeted genotyping amplicons are generated by addition of primers that flank mutations of interest, enabling parallel amplification of these loci from gDNA and, optionally, also from cDNA. This is a critical step in the protocol. It is important to work quickly under sterile and PCR-free conditions to prevent mRNA degradation. We recommend working with 1-2 plates at a time, increasing up to 4 plates at a time once confident with the protocol.

**CRITICAL:** Prepare all reagents beforehand, including single-use TSO-LNA aliquots, and targeted cDNA and gDNA genotyping primers.

**CRITICAL:** Perform cell lysis, protease heat inactivation, RT, and addition of PCR enzyme mix in a dedicated pre-PCR clean area, ideally in a biosafety cabinet, to avoid contamination from PCR products and RNases. Clean the biosafety cabinet and pipets with a cleaning agent to remove RNases (e.g., RNaseZap) and use RNase-free filter tips. Once the PCR mix has been added, the PCR should be carried out in a post-PCR area.

27. Take the sorted plates (point 26) and TSO-LNA from the −80°C freezer and keep on dry ice until ready to perform the cell lysis and heat inactivation.
28. Thaw reagents needed for the RT master mix, except the TSO-LNA and Maxima H-minus RT enzyme, and place on a cool rack or on ice. Cool down a 384-well cold rack to 4°C.
29. Prepare the MANTIS Liquid Handling Platform for dispensing the RT mix by performing initialization and cleaning with RNase away.
30. Preheat a thermocycler to 72°C with the lid heated to 105°C.
31. Perform the cell lysis and protease heat inactivation:
  a. Take the plate containing sorted cells from dry ice and place directly on the thermocycler.
  b. Incubate at 72°C for 15 min. This inactivates the protease to prevent it interfering with subsequent enzymatic steps.
32. During the cell lysis and protease heat inactivation, prepare the RT master mix:

**Table:** RT master mix

Reagent	Reaction concentration	Volume per well (μL)	Volume for 192 wells + 15% dead volume (μL)
Tris-HCl pH 8.3 (1 M)	25 mM	0.1	22
NaCl (1 M)	30 mM	0.12	26.4
MgCl2 (100 mM)	2.5 mM	0.1	22
GTP (100 mM)	1 mM	0.04	8.8
DTT (100 mM)	8 mM	0.32	70.4
Nuclease-free water		Variable	Variable
Targeted cDNA genotyping primers (25 μM)	70 nM per primer	0.0112 per primer	2.5 per primer
RNase Inhibitor (40 U/μL)	0.5 U/μL	0.05	11
TSO-LNA (100 μM)	2 μM	0.08	17.6
Maxima H-minus RT enzyme (200 U/μL)	2 U/μL	0.04	8.8
**Total**		**1.0**	**220**
**TOTAL (Cumulative)**		**4.0**

***Note:*** Addition of targeted cDNA genotyping primers in this step may improve the efficiency of genotyping. However, this is not essential, and users may choose to omit these if they find that these primers interfere with cDNA amplification when performing validation experiments. For example, if users notice that the quality of the cDNA traces in validation experiments improves substantially when cDNA genotyping primers are omitted (decreased concatemerization or increased cDNA yield), cDNA genotyping primers can be omitted. In our experiments, the addition of cDNA genotyping primers increased the rates of correct genotyping by ∼5%, but most cells are successfully genotyped using gDNA genotyping primers alone.

**CRITICAL:** Proceed immediately with the next step once heat inactivation is complete to avoid mRNA degradation.

33. Dispense the RT master mix:
  a. Remove the plate from the thermocycler, centrifuge in a plate spinner at 500 × g for 15 s and place on a cold rack.
  b. Carefully remove the plate seal.
  c. Dispense 1 μL of RT master mix into each well of the plate, using the MANTIS with a High Volume (HV) chip (Figure 11).
34. Carefully seal the plate with a plastic PCR film (MicroAMp Clear Adhesive Film, Thermo Fisher Scientific, Cat# 4306311), spin down at 500 × g for 15 s, and immediately place on the thermocycler to run the RT program:

**Table:** RT cycling conditions

Temperature	Time	Cycles
42°C	90 min	1
50°C	2 min	10 cycles
42°C	2 min	
85°C	5 min	1
4°C	Hold

**CRITICAL:** This step should take no longer than 5–6 min; otherwise, mRNA degradation will occur!

35. Start preparing the pre-amplification PCR mix when the RT program is nearly finished. Thaw reagents required for the PCR mix and place on a cool rack or ice. Cool down a 384-well cold rack to 4°C.
36. Prepare the MANTIS for dispensing the PCR mix by performing initialization and cleaning with RNase away.
37. Prepare the PCR master mix by combining the following components:

**Table:** Pre-amplification PCR master mix

Reagent	Reaction concentration	Volume per well (μL)	Volume for 192 wells + 9% dead volume (μL)
Kapa HiFi HotStart Ready Mix (2×)	1×	5.00	1050
ISPCR primer (10 μM)	50 nM	0.05	10.5
Nuclease-free water		Variable	Variable
Targeted cDNA genotyping primers (25 μM)	28 nM per primer	0.0112 per primer	2.35 per primer
Targeted gDNA genotyping primers (100 μM)	400 nM per primer	0.04 per primer	8.4 per primer
**Total**		**6.0**	**1260**
**TOTAL (Cumulative)**		**10.0**

38. Dispense the PCR master mix:
  a. Remove the plate from the thermocycler, centrifuge in a plate spinner at 500 × g for 15 s and place on a cold rack.
  b. Carefully remove the plate seal.
  c. Dispense 6 μL of PCR master mix into each well of the plate using the MANTIS with a High Volume (HV) chip.
39. Perform the Pre-amplification PCR:
  a. Carefully seal the plate with a plastic PCR film and spin down at 500 × g for 15 s.
  b. Place the plate on ice, and transfer to a dedicated post-PCR area.
  c. Place the plate on a thermocycler and run the PCR program:

**Table:** Pre-amplification PCR cycling conditions

Steps	Temperature	Time	Cycles
Initial Denaturation	98°C	3 min	1
Denaturation	98°C	20 sec	18-25 (21 for HSPCs)
Annealing	67°C	30 sec	
Extension	72°C	6 min	
Final extension	72°C	5 min	1
Hold	4°C	Hold

***Note:*** The PCR cycle number depends on the input and is cell-type specific (preparation 1).

**Pause point:** Once the PCR has finished, spin down at 500 × g for 30 s, and snap freeze on dry ice. Frozen plates can be stored at −20°C for up to 6 months.

### Part 4: Pooling, cDNA-amplicon mix dilution, and cDNA quality control

**Timing: 2 h**

**Figure 13:**
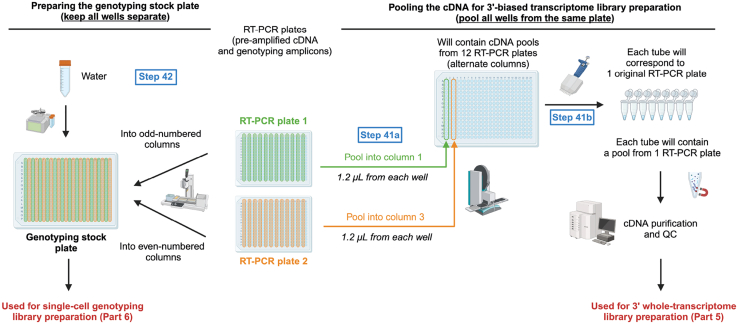
cDNA pooling and genotyping stock plate preparation After PCR, a portion of the material from each plate is transferred into a new genotyping stock plate containing nuclease-free water (top). This plate will be used for single-cell genotyping library construction. The remaining material from each well of a plate is pooled for cDNA purification, keeping each plate separate (bottom).

This section describes how to pool and purify scRNA-seq libraries and make dilutions of the cDNA-amplicon mix to use for single-cell genotyping (Figure 13). First, we pool an aliquot of the RT-PCR product from each well of each plate to generate a cDNA pool that is used for transcriptome library preparation (part 5). Second, we transfer part of the material from the original RT-PCR plates to fresh plates, which will serve as genotyping stocks for the generation of single-cell genotyping libraries (part 6). The genotyping PCR steps will be performed in plate format to fully barcode the genotyping amplicons.

***Note:*** Following RT-PCR, pre-amplified cDNA contains a 3′ cell barcode, unique to each well on the plate. cDNA libraries from each plate can therefore be pooled together for subsequent transcriptome library preparation (part 5). However, targeted genotyping amplicons are not yet barcoded; therefore, care must be taken not to cause cross-contamination between wells.

40. Thaw the RT-PCR plate(s) containing the pre-amplified cDNA-amplicon mix from point 39 and spin down at 3,000 × g for 1 min at 21°C.

***Note:*** Take care that there are no droplets on the PCR film when removing the film as this may give rise to cross-contamination between wells.

41. For each RT-PCR plate, pool 1.2 μL from every well, following these steps:
  a. Pool 1.2 μL from every well in each row of the RT-PCR plate into one column of a new PCR plate (Figure 13) using a Mosquito (SPT Labtech).
    ***Note:*** A single column of the new pooling plate should contain material from one entire RT-PCR plate, with each well containing material from one row of the RT-PCR plate.
  b. Repeat this for all RT-PCR plates, with each new RT-PCR plate being transferred to a new column of the fresh plate.
    **CRITICAL:** Genotyping amplicons are not yet barcoded. Avoid any cross-well contamination while pooling cDNA. Therefore, exchange Mosquito tips between every column of the RT-PCR plate and between RT-PCR plates.
    ***Note:*** We perform this step using a Mosquito; however, this step can also be done manually or adapted to an Integra VIAFLO or Biomek FxP (Beckman Coulter) liquid handling platforms.
  c. Manually pool the cDNA libraries from each column of the pooling plate (point 41a) into a single Eppendorf or PCR tube, such that each final pool contains libraries from one original RT-PCR plate. These cDNA pools will be used in point 43.
    **CRITICAL:** Keep pooled cDNA libraries separate for each plate. Plate indexes will be added at a later stage.
42. Using the remaining non-pooled material from the original RT-PCR plates, prepare the genotyping stock plates which will be used for single-cell genotyping library preparation, following these steps:
  a. Prepare a fresh 384-well plate.
  b. Aliquot 10 μL of nuclease-free water into each well of this 384-well plate using the MANTIS with a HV chip.
  c. Transfer 6 μL of non-pooled material from the original RT-PCR plate (containing cDNA and non-barcoded genotyping amplicons from 192 cells) into odd-numbered columns of the fresh plate.
    ***Note:*** We perform this step using the Integra VIAFLO.
  d. Transfer 6 μL of non-pooled material from a second RT-PCR plate into even-numbered columns of the genotyping stock plate.
    ***Note:*** We perform this step using the Integra VIAFLO.
    ***Note:*** The genotyping stock plate now contains material from 384 cells. When cells were sorted into alternate columns of the RT-PCR plate, combining the material from two RT-PCR plates into a single genotyping stock plate makes the downstream genotyping library preparation more efficient. To transfer half 384-well plates, either use a 96-well VIAFLO pipetting head, or split your tips in two boxes and use a 384-well VIAFLO pipetting head, taking 192 tips at a time.
    **CRITICAL:** Only combine material from two original RT-PCR plates into a single genotyping stock plate if the same amplicons are being genotyped. Make note of which columns contain samples from which original RT-PCR plate.
  e. Repeat steps 42a–42d for all RT-PCR plates. Hence, the number of genotyping stock plates will be half the number of RT-PCR plates. Genotyping stock plates will be used in part 6.
    **Pause point:** Seal and spin down genotyping stock plates at 500 × g for 30 s, and snap freeze on dry ice. Frozen plates can be stored at −20°C for up to 6 months. You may now proceed with bead purification of the pooled cDNA libraries or freeze them and perform bead clean-up later.
    **CRITICAL:** When freezing plates, use aluminum seals, as plastic seals may detach in the freezer. It is important to spin down and snap freeze plates on dry ice to avoid liquid freezing on the sides of the wells or the cover, which may lead to cross-contamination between wells.
43. Purify the pooled cDNA libraries, keeping the material from each plate separate, as follows:
  a. Prior to starting, place the AMPure XP beads at 21°C for 30 min. Vortex thoroughly to ensure that the beads are properly mixed with the buffer.
  b. Prepare 80% ethanol solution in nuclease-free water.
  c. Dispense 66 μL of AMPure XP beads into wells of a V-bottom 96-well plate (number of wells equal to the number of cDNA pools to be purified).
  d. Add 110 μL of pooled cDNA (from point 41b) to the AMPure XP beads (0.6:1 beads to cDNA ratio) and pipette to mix. Avoid bubbles. Keep the cDNA pool from each original RT-PCR plate separate.
  e. Incubate for 5 min at 21°C.
  f. Place the plate on a 96-well magnetic stand and incubate for 2 min until the liquid is clear of beads.
  g. Carefully remove the supernatant with a pipette.
  h. Wash the beads twice by adding 200 μL 80% ethanol to each well, incubating for 30 s and then removing and discarding the supernatant, taking care not to disturb the beads.
  i. After the second wash, let the beads air-dry for 3 min. The beads are dry enough when the surface of the pellet changes from shiny to matt.
    **CRITICAL:** Be careful not to over-dry the beads as this makes it difficult to resuspend them and may reduce cDNA yield.
  j. Remove the plate from the magnet, resuspend beads in 110 μL EB buffer and mix thoroughly by pipetting.
  k. Incubate for 5 min.
  l. Place the plate back on the magnet and wait for 2–3 min for the supernatant to be completely clear of beads.
  m. Transfer the supernatant containing 110 μL of purified product from point 43l to 66 μL of freshly aliquoted AMPure XP beads (0.6:1 beads to cDNA ratio) and pipette to mix.
  n. Incubate for 5 min at 21°C.
  o. Place the plate on the magnetic stand and incubate for 2 min until the liquid is clear of beads.
  p. Carefully remove the supernatant with a pipette.
  q. Wash the beads twice by adding 200 μL 80% ethanol to each well, incubating for 30 s and then removing and discarding the supernatant. After the second wash, carefully remove any residual ethanol using 20 μL tips.
  r. Let the beads air-dry for 3–5 min. The beads are dry enough when the surface of the pellet changes from shiny to matt.
    **CRITICAL:** Be careful not to over-dry the beads as this makes it difficult to resuspend them and may reduce cDNA yield. However, it is also important to remove all residual ethanol.
  s. Remove the plate from the magnet. Resuspend beads in 25 μL EB buffer and mix thoroughly by pipetting.
  t. Incubate for 5 min to elute cDNA.
  u. Place the plate back on the magnet and wait for 2–3 min for the supernatant to be completely clear of beads.
  v. Transfer the supernatant containing purified cDNA to a new plate, PCR strips, or LoBind Eppendorf tubes.
44. Check cDNA quality by capillary electrophoresis, such as using a Bioanalyzer (High Sensitivity DNA Kit, Cat# 5067-4626; Agilent), Fragment Analyzer (High Sensitivity NGS Fragment Analysis Kit (1–6,000 bp); Agilent) or TapeStation (High Sensitivity D5000 ScreenTape and Reagents; Agilent). Good quality cDNA libraries are shown in Figure 2. Troubleshooting 1, 2, 3, and 4
45. Quantify the cDNA concentration using Qubit dsDNA HS Assay Kit. Troubleshooting 1.

**Pause point:** The purified cDNA can be stored at −20°C for at least 1 year.

### Part 5: Whole-transcriptome library preparation and sequencing

**Timing: 5 h**

**Figure 14:**
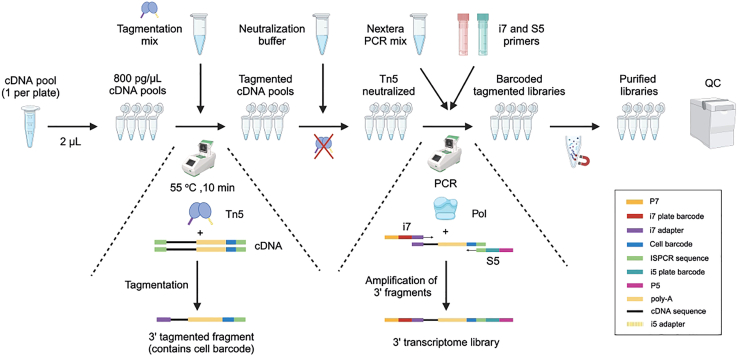
Construction of 3′ biased whole-transcriptome libraries Above, schematics of tagmentation followed by PCR. Below, schematics of the chemistry associated with each step. Sequence color-coding in the bottom right corner.

In this section, we describe how to prepare whole transcriptome libraries from pooled pre-amplified cDNA. First, the purified cDNA is tagmented using the Tn5 enzyme from the Nextera XT DNA Library Prep Kit. The 3′ fragments of the tagmented cDNA are then amplified and indexed using custom P5 primers that bind to the 3′ adapter and Nextera i7 index adapters, leading to the generation of 3′ biased libraries with a cell-specific barcode (added via the oligo(dT) during RT-PCR) and a plate-specific i7 index. Finally, amplified 3′ transcriptome libraries are purified, pooled, and sequenced (Figure 14).

***Note:*** The tagmentation-based strategy for generating 3′ transcriptome libraries (Figure 14) is based on the original TARGET-seq protocol^3^ with a few modifications. Specifically, we increased the input of cDNA into the tagmentation reaction to increase library complexity and modified the P5 adapter to incorporate i5 plate indexes into the 3′ cDNA library. The addition of i5 indexes introduces redundancy between the i7 and i5 plate indices which mitigates against read misassignment resulting from index hopping.

46. Normalize the cDNA concentration of each purified cDNA pool to 800 pg/μL as follows:
  a. Calculate the volume of water needed to dilute 2 μL of each purified cDNA pool to 800 pg/μL.
  b. Prepare a PCR tube for each purified cDNA pool and add the calculated volumes of nuclease-free water from Step 46a to each well.
  c. Add 2 μL of each purified cDNA pool to each well and mix.
47. Prepare the tagmentation mix according to the number of cDNA pools to be tagmented:

**Table:** Tagmentation mix

Reagent	Volume per reaction (μL)
Tagment DNA (TD) buffer	10.0
Amplicon tagment mix (ATM)	5.0
**Total**	**15.0**

48. Dispense 15 μL of the Tagmentation Mix into PCR tubes or a 96-well plate.
49. Add 5 μL of normalized 800 pg/μL cDNA (Step 46) to the Tagmentation Mix, keeping each pool in a separate reaction, and pipette to mix.
50. Incubate on a thermocycler at 55°C for 10 min to tagment the cDNA.
51. Add 5 μL Neutralize Tagment (NT) buffer and pipette mix to terminate the tagmentation reaction.

***Optional:*** Alternatively, if there is not sufficient NT buffer, 5 μL of 0.2% SDS may be used to neutralize the tagmentation reaction.

52. Add 5 μL of 2 μM i7 index primer and 5 μL of 2 μM custom i5 index primer (Table S2) to each well containing tagmented cDNA.

***Note:*** The custom i5 index primer binds the barcoded oligo(dT)-ISPCR adapter, while the i7 index primer binds adapters introduced by the Tn5 enzyme, resulting in amplification of the 3′ fragments containing the cell barcode.

**CRITICAL:** i7 and i5 indexes are used for demultiplexing reads from different cDNA pools. Ensure that each cDNA pool has a unique i7/i5 combination. Take care to choose indexes that achieve color balance on Illumina systems to ensure successful sequencing. This is particularly important when using ≤ 8 different i7 or i5 indexes in a sequencing run.

***Optional:*** Note that the use of i5 barcodes is optional and a generic custom i5 can be used instead. Theoretically, the i7 barcodes may be sufficient to act as plate barcodes. However, using i5 indexes increases multiplexing capacity. Moreover, if multiple i5 indexes are used, this reduces index hopping by adding redundancy to the plate indexes.

53. Add 15 μL of the Nextera PCR Master Mix (NPM) to each well and pipette to mix. Incubate on a thermocycler using the following PCR program:

**Table:** Tagmentation PCR cycling conditions

Steps	Temperature	Time	Cycles
Initial Denaturation	95°C	30 sec	1
Denaturation	95°C	10 sec	13 cycles
Annealing	55°C	30 sec	
Extension	72°C	30 sec	
Final extension	72°C	5 min	1
Hold	4°C	Hold

**Figure 15:**
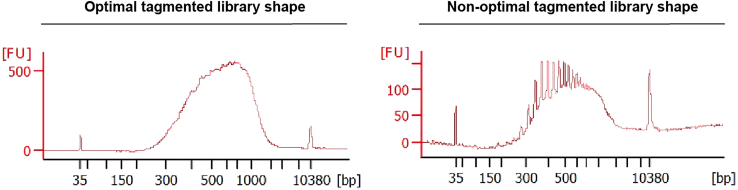
Optimal and non-optimal tagmented cDNA library Left, an optimal tagmented cDNA library shows a smooth profile with the highest DNA concentration in the range between 400 and 1,000 bp. Right, a non-optimal tagmented cDNA library shows periodic spikes (‘hedgehog’ pattern) indicative of TSO concatemerization.
  b. If there are traces of primer dimers, repeat the bead purification. Troubleshooting 5, 6, and 7.
56. Quantify the cDNA concentration using Qubit dsDNA HS Assay Kit and calculate the molarity using the fragment size measured by capillary electrophoresis.
57. Pool equimolar concentrations of each library in a microfuge tube and dilute to 4 nM using Qubit and Bioanalyzer readouts. Use Table S4 as template.

***Note:*** Libraries labeled with different i7 and/or i5 indexes can be multiplexed and sequenced together.

**Figure 16:**
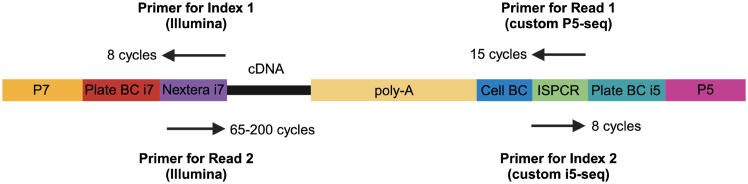
Final 3′ cDNA fragment ready for sequencing The sequencing primers used are shown above and below the fragment. The number of cycles for each read and index are shown next to the respective primers. Note that the use of the custom i5-seq primer is optional.

54. Purify the amplified and tagmented cDNA libraries:
  a. Prior to starting, place the Ampure XP beads at 21°C for 30 min. Vortex thoroughly to ensure that the beads are properly mixed with the buffer.
  b. Prepare 80% ethanol solution in nuclease-free water.
  c. Add 35 μL of AMPure XP beads to each sample of tagmented library (0.7:1 beads to cDNA ratio) and pipette mix.
  d. Incubate for 5 min at 21°C.
  e. Place the tubes on a magnetic stand and incubate for 2 min until the liquid is clear of beads.
  f. Carefully remove the supernatant.
  g. Wash the beads twice by adding 100 μL of 80% ethanol to each well, incubating for 30 sec and then removing and discarding the supernatant. After the second wash, remove any residual ethanol using 20 μL tips.
  h. Let the beads air-dry for 3–5 min. The beads are dry enough when the surface of the pellet changes from shiny to matt.
  i. Remove the tubes from the magnet, resuspend beads in 35 μL EB buffer and mix thoroughly by pipetting.
  j. Incubate for 5 min.
  k. Place the plate back on the magnet and wait for 2–3 min for the supernatant to be completely clear of beads.
  l. Transfer the supernatant containing 35 μL of purified product from point 54k to 24.5 μL of freshly aliquoted AMPure XP beads and pipette to mix.
  m. Incubate for 5 min at 21°C.
  n. Place the plate on the magnetic stand and incubate for 2 min until the liquid is clear of beads.
  o. Carefully remove the supernatant with a pipette.
  p. Wash the beads twice by adding 100 μL 80% ethanol to each well, incubating for 30 sec, and then removing and discarding the supernatant. After the second wash, carefully remove any residual ethanol using 20 μL tips.
  q. Let the beads air-dry for 3–5 min. The beads are dry enough when the surface of the pellet changes from shiny to matt.
    **CRITICAL:** It is important to remove all residual ethanol, given that traces of ethanol can interfere with sequencing. Be careful not to over-dry the beads as this makes it difficult to resuspend them and may reduce cDNA yield.
  r. Remove the tubes from the magnet, resuspend the final purified libraries in 20 μL of EB buffer and mix thoroughly by pipetting.
  s. Incubate for 3 min to elute cDNA.
  t. Place the plate back on the magnet and wait for 2–3 min for the supernatant to be completely clear of beads.
  u. Transfer the supernatant containing purified cDNA to a new plate or Eppendorf tubes.
    **Pause point:** The purified tagmented cDNA libraries can be stored at −20°C for at least 2 months.
55. Check the size distribution of the tagmented libraries quality by capillary electrophoresis, such as using a Bioanalyzer (High Sensitivity DNA Kit, Cat# 5067-4626; Agilent), Fragment Analyzer (High Sensitivity NGS Fragment Analysis Kit (1–6,000 bp); Agilent) or TapeStation (High Sensitivity D5000 ScreenTape and Reagents; Agilent).
  a. The average fragment size should be 600–850 bp. An example of a good and a poor quality library is shown in Figure 15.

58. Sequence the libraries (Figure 16) on an Illumina platform (NextSeq, HiSeq, or NovaSeq):
  a. Load 300 nM of a custom sequencing primer for Read 1 and, if using i5 indexes, for Index Read 2.
  b. Use the following sequencing configuration:

**Table:** 

Read	Sequencing primer	Cycles
Read 1 (cell barcode)	P5-SEQ custom primer	15
Index 1 (plate barcode)	Illumina primers	8
Read 2 (cDNA)	Illumina primers	65–200
Index 2 (plate barcode; if using i5 indexes)	I5-SEQ custom primer	8

**CRITICAL:** Do not use more than 15 cycles for Read 1. Using more than 15 cycles with cause the sequencing to run into the poly(A) tail, leading to a lack of color diversity which may cause the sequencing run to fail.

### Part 6: Single-cell genotyping library preparation

**Timing: 5 h for 1 plate, 2 days for 5–20 genotyping plates, and 3 days for 20–40 genotyping plates**

In this section, we describe how to generate Illumina-compatible libraries for single-cell genotyping, starting from the genotyping stock plates containing diluted cDNA-amplicon mix prepared in part 4, point 42.

**Figure 17:**
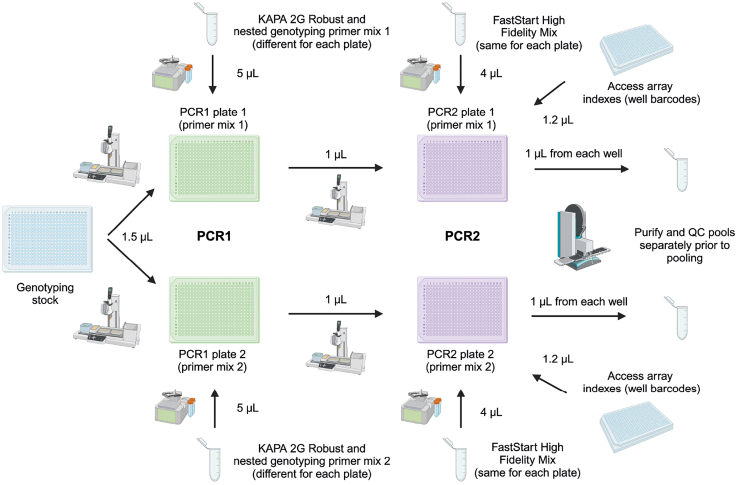
Schematics of single-cell genotyping library preparation In this example, 2 genotyping primer mixes are used. First, 5 μL of PCR1 mix, containing enzyme and barcoded nested genotyping primers, are dispensed into a fresh 384-well plate (PCR1 plate). A different PCR1 mix is aliquoted into each PCR1 plate, so that each plate contains primers with a unique plate barcode. Then, 1.5 μL of material is transferred from the genotyping stock plate into the PCR1 plate, keeping material from each well separate, and the PCR1 reaction is performed. Next, the PCR2 reaction is set up by transferring 1.2 μL of 384 barcoded Access array indexes (well barcodes) into fresh PCR2 plates using the VIAFLO, followed by the addition of 4 μL of the generic PCR2 mix using the Mantis. When PCR1 is finished, 1 μL of material is transferred from the PCR1 plate into the PCR2 plate, keeping material from each well separate, and the PCR2 reaction is performed.

***Note:*** For each cell, non-barcoded genotyping amplicons were generated during the pre-amplification PCR (part 3) by addition of target-specific genotyping primers. Now, genotyping amplicons need to be further enriched and fully barcoded, to be able to assign them to single cells. This is achieved via two additional PCR steps (Figures 3 and 17). In the first PCR (genotyping PCR1), target-specific primers that are nested within the original amplicon(s) containing universal CS1 (forward primer) and CS2 (reverse primer) adapters are used to amplify target regions of interest. Plate-specific 6 bp barcode sequences are incorporated into these primers, between the CS adapters and the target-specific sequence (Table S5), enabling libraries from different plates to be pooled together later (Table S4). In the second PCR (genotyping PCR2), primers that bind to the CS1/CS2 adapters (from the Access Array Barcode Library for Illumina Sequencers-384, Single Direction kit) are used to attach Illumina-compatible sequencing adapters to genotyping amplicons. The primer that binds to the CS2 adapter also contains a 10 bp index, serving as cell barcode, which is well-specific. The combination of plate and cell barcode is unique for each cell across all plates, allowing all amplicons to be sequenced together. After genotyping PCR2, libraries are pooled, purified, and quantified for sequencing.

***Note:*** The steps described in this section are performed using the MANTIS, INTEGRA VIAFLO, and the Mosquito HTS Nanoliter Liquid Handler. However, users may automate the protocol on other liquid handling platforms, such as the Biomek FxP Liquid Handling Platform.

***Note:*** Genotyping PCR1 is performed with a separate reaction for each cell. Users may need to perform several genotyping PCR1 reactions per cell (Table S6; Figure 17), using different primer mixes, depending on the number of loci genotyped and other factors listed below. PCR1 reactions with different primer mixes are performed on separate plates. We suggest performing separate reactions for loci in lowly and highly expressed genes. cDNA amplicons from highly expressed genes can be amplified with gDNA amplicons, whereas cDNA amplicons from lowly expressed genes should be amplified in a separate reaction, so they are not outcompeted. Care should be taken to avoid combining gDNA and cDNA primers that will interfere with each other during parallel amplification (e.g. if one primer binds within the other amplicon), in which case these should be amplified in separate reactions. Finally, amplicons with substantially different amplification efficiencies should also be amplified in separate reactions, to avoid the lower abundance amplicon being outcompeted, as this can result in poor coverage during sequencing.

***Note:*** Remember that the genotyping stock plate contains material from 2 original sorted plates.

59. Thaw barcoded genotyping PCR1 primers, prepare the genotyping PCR1 primer mix for each plate, and each PCR1 primer combination (Table S6). The tables below show an example with two separate PCR1 primer mixes:

**Table:** Genotyping PCR1 Primer mix 1 (for example, 3 gDNA primer pairs)

Reagent	Volume per reaction (μL)	Volume for 384-well plate + 15% dead volume (μL)
Primer 1 (100 μM)	0.0195	8.58
Primer 2 (100 μM)	0.0195	8.58
Primer 3 (100 μM)	0.0195	8.58
Primer 4 (100 μM)	0.0195	8.58
Primer 5 (100 μM)	0.0195	8.58
Primer 6 (100 μM)	0.0195	8.58
RT-PCR Grade Water	1.63	719
**Total**	**1.75**	**770**

**Table:** Genotyping PCR1 Primer mix 2 (for example, 2 cDNA primer pairs)

Reagent	Volume per reaction (μL)	Volume for 384-well plate + 15% dead volume (μL)
Primer 1 (100 μM)	0.0195	8.58
Primer 2 (100 μM)	0.0195	8.58
Primer 3 (100 μM)	0.0195	8.58
Primer 4 (100 μM)	0.0195	8.58
RT-PCR Grade Water	1.67	736
**Total**	**1.75**	**770**

***Note:*** Work in a designated pre-PCR area when preparing genotyping PCR1 primers to avoid contamination with PCR products.

**CRITICAL:** All primers used to amplify targets from each initial cDNA-amplicon stock plate need to have the same plate barcode, even if they are not all in the same genotyping PCR1 reaction. Take care to label each PCR1 primer mix with the plate barcode. If barcoded primers are added to the wrong mix, genotyping amplicon reads will be assigned to the wrong plate.

60. Thaw the genotyping stock plates (part 4, point 42) by centrifuging at 3000 × g for 3 min at 21°C.

**CRITICAL:** Make sure to centrifuge plates at 21°C, because centrifuging at 4°C will cause condensation on the PCR film, which may lead to cross-contamination between wells. Take care when removing PCR film to avoid cross-contamination.

61. Prepare to set up genotyping PCR1:
  a. Clean working surfaces with 70% ethanol.
  b. Thaw KAPA2G Robust HS Ready Mix and keep on ice.
  c. Vortex genotyping PCR1 primer mixes and keep on ice.
  d. Prepare sufficient fresh 384-well plates for the number of genotyping PCR1 reactions.
62. Prepare Genotyping PCR1 Master Mix for the first PCR1 plate (containing a specific primer mix and a unique plate barcode) and pipette mix 10 times:

**Table:** Genotyping PCR1 Master mix

Reagent	Volume per reaction (μL)	Volume for 384-well plate + 10% dead volume (μL)
KAPA 2G Robust HS ready mix	3.25	1373
Genotyping PCR1 primer mix	1.75	739
**Total**	**5.0**	**2112**

63. Dispense 5 μL of Genotyping PCR1 Master Mix into each well of a fresh genotyping PCR1 384-well plate, using the MANTIS with the HV chip.
64. Transfer 1.5 μL from the genotyping stock plate into the genotyping PCR1 plate and pipette mix 3 times, using the VIAFLO.
65. Perform genotyping PCR1:
  a. Seal the genotyping stock plate with an aluminum adhesive seal and the genotyping PCR1 plate with a plastic PCR adhesive seal.
  b. Centrifuge both plates in a plate spinner for 10 s at 500 × g.
  c. Snap freeze the genotyping stock plate on dry ice.
  d. Place the genotyping PCR1 plate on a thermocycler with the lid heated at 105°C and run the following PCR1 program:

**Table:** Genotyping PCR1 Cycling conditions

Steps	Temperature	Time	Cycles
Initial Denaturation	95°C	3 min	1
Denaturation	95°C	15 sec	20 cycles
Annealing	60°C	20 sec	
Extension	72°C	1 min	
Final extension	72°C	5 min	1
Hold	4°C	Hold

66. Repeat points 62–65 for each PCR1 primer mix.

**CRITICAL:** If dispensing enzyme-primer master mix using a microfluidic dispenser such as the MANTIS, ensure that the fluidics are thoroughly cleaned with ethanol and nuclease-free water between each master mix to avoid carry-over of barcoded primers from one plate to another. Carry-over of barcoded primers will lead to reads being misassigned to cells from the wrong plate.

**Pause point:** When genotyping PCR1 is complete, you can proceed directly with genotyping PCR2, or you can snap freeze genotyping PCR1 plates and store them at −20°C for at least 3 months.

***Note:*** Genotyping PCR2 (Figure 17) is performed with a separate reaction for each cell. Where several PCR1 reactions (with different primer mixes) were performed for each cell, one PCR2 reaction will be performed for each PCR1 reaction.

67. Prepare plates for genotyping PCR2 containing 1.2 μL of 2 μM barcoded PCR2 primers:
  a. Prepare fresh 384-well plates (equal to the number of genotyping PCR1 plates).
  b. Thaw the Access Array 2 μM stock plate (preparation 5) and centrifuge it at 3,000 × g for 3 min at 21°C.
  c. Aliquot 1.2 μL of material from the Access Array 2 μM stock plate into each fresh 384-well plate, using the VIAFLO.
  d. Cover with adhesive aluminum seals, centrifuge in a plate spinner for 10 s at 500 × g, and place on ice.

**CRITICAL:** Aliquoted plates need to be covered and kept on ice to avoid evaporation.

**Pause point:** You can either proceed with genotyping PCR2, or you can snap freeze these plates on dry ice and store them at −20°C for at least 3 months.

68. Prepare to set up the genotyping PCR2:
  a. Clean work surfaces with 70% ethanol.
  b. Thaw the genotyping PCR1 plates (point 66) by centrifuging them at 3000 × g for 3 min at 21°C.
  c. Centrifuge the genotyping PCR2 plates containing 1.2 μL of 2 μM barcoded PCR2 primers (point 67) at 3,000 × g for 2 min at 21°C.
  d. Thaw FastStart High Fidelity PCR reagents and keep on ice, except DMSO, which should be kept at 21°C.
69. Prepare the genotyping PCR2 master mix and pipette mix 10 times:

**Table:** Genotyping PCR2 Master mix

Reagent	Volume per reaction (μL)	Volume for 384-well plate + 4% dead volume (μL)
FastStart Buffer 10× (labeled 3)	0.6 μL	240 μL
MgCl2 (labeled 4)	1.08 μL	432 μL
DMSO (labeled 5)	0.3 μL	120 μL
Nucleotide mix (labeled 6)	0.12 μL	48 μL
Nuclease-free water	1.84 μL	736 μL
Enzyme (labeled 1)	0.06 μL	24 μL
**Total**	**4.0**	**1600**

70. Dispense the genotyping PCR2 master mix into the genotyping PCR2 plate containing 1.2 μL of 2 μM barcoded PCR2 primers:
  a. Dispense 4 μL of the genotyping PCR2 master mix into each well of the genotyping PCR2 plate using the MANTIS with a HV chip.
  b. Seal the plate and centrifuge in a plate spinner for 10 s at 500 × g.
71. Transfer 1 μL of material from each well of the genotyping PCR1 plate into the genotyping PCR2 plate and pipette mix 3 times, using the VIAFLO.
72. Perform genotyping PCR2:
  a. Seal the genotyping PCR1 plate with an aluminum adhesive seal and the genotyping PCR2 plate with a plastic PCR adhesive seal.
  b. Centrifuge both plates in a plate spinner for 10 s at 500 × g.
  c. Snap freeze the genotyping PCR1 plate on dry ice.
  d. Place the genotyping PCR2 plate on a thermocycler with the lid heated at 105°C and run the following PCR2 program:

**Table:** Genotyping PCR2 cycling conditions

Steps	Temperature	Time	Cycles
Initial Denaturation	95°C	10 min	1
Denaturation	95°C	15 sec	10 cycles
Annealing	60°C	30 sec	
Extension	72°C	1 min	
Final extension	72°C	5 min	1
Hold	4°C	Hold

73. Repeat points 69–72 for all genotyping PCR2 plates.

***Optional:*** If two people are working, we suggest that one person operates the MANTIS while the other operates the VIAFLO. This setup will drastically speed up the execution of the protocol. We usually process 20–40 genotyping PCR2 plates per day using this setup.

**Pause point:** We recommend preparing all genotyping PCR2 plates before proceeding to the next step. When genotyping PCR2 is complete, you can proceed, or you can snap freeze genotyping PCR2 plates on dry ice and store them at −20°C for at least 3 months.

***Note:*** Each well of each genotyping PCR2 plate is now fully barcoded with a combination of well and plate barcode. However, we pool and purify each genotyping PCR2 plate separately for QC purposes.

**Figure 18:**
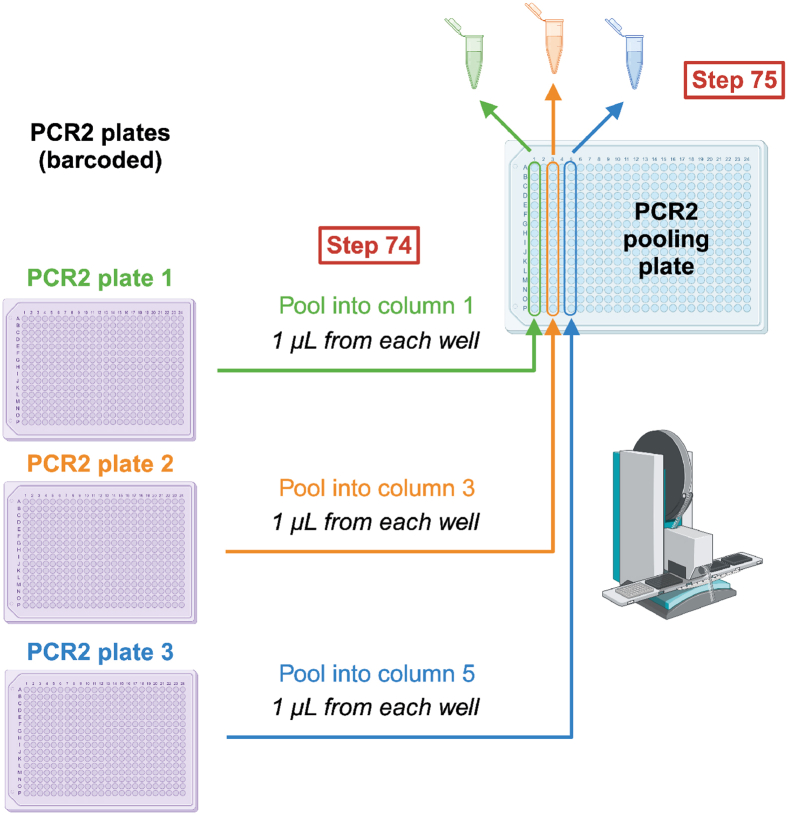
Pooling genotyping PCR2 plates Each PCR2 plate is pooled into a single column of a fresh PCR2 pooling plate, using the Mosquito. These columns are then pooled into separate tubes (one tube per original PCR2 plate).
  d. When the plate is complete, cover the genotyping PCR2 plate with an aluminum adhesive seal and snap freeze on dry ice.
  e. Repeat points 74a–d for all genotyping PCR2 plates, pooling into alternate columns of the PCR2 pooling plate, so that each column will contain material from one full genotyping PCR2 plate.
75. Pool the material from each column of the PCR2 pooling plate into a 1.5 mL microcentrifuge tube, labeled according to the genotyping PCR2 plate, using a P200.

***Note:*** Each tube should contain the PCR2 material from one genotyping PCR2 plate.

**Figure 19:**
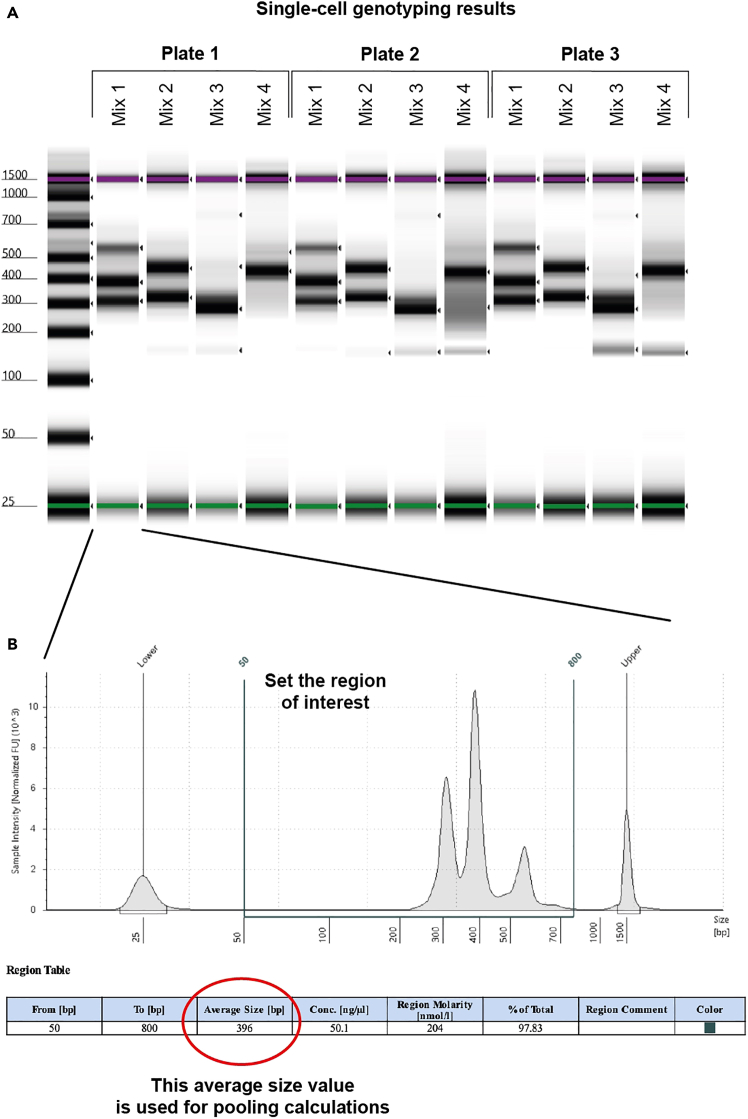
Optimal single-cell genotyping libraries (A) In this example, 4 genotyping primer mixes were used for each original plate. Each mix derives from a pool of 190 single cells. Each reaction shows bands of the expected size. (B) Detailed inspection of the first mix from A. The region encompassing the bands of interest needs to be set in order to estimate the average size (in bp) for each mix. This value will be used for subsequent pooling.

***Note:*** Barcodes and adapter sequences add a total of 115 extra bp to the genotyping amplicon.

**Figure 20:**
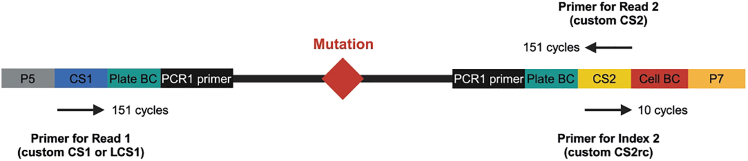
Final genotyping fragment ready for sequencing The custom sequencing primers used are shown above and below the fragment. The number of cycles for each read and index are shown next to the respective primers.

74. Pool the 384 libraries from each genotyping PCR2 plate (1 μL from each well):
  a. Centrifuge all genotyping PCR2 plates in a plate spinner for 2 min at 3000 × g and keep them on ice.
  b. Prepare a fresh 384-well plate, and label it as “PCR2 pooling plate”.
  c. Pool 1 μL from every well in each row of the genotyping PCR2 plate into one column of the PCR2 pooling plate using the Mosquito HTS Nanoliter Liquid Handler (Figure 18).
    ***Note:*** A single column of the PCR2 pooling plate should contain material from the entire PCR2 plate, with each well containing material from one row of the PCR2 plate.
    ***Note:*** There is no need to exchange tips between each column of the PCR2 plate, as amplicons are now fully barcoded.

76. Purify the genotyping PCR2 libraries:
  a. Prior to starting, place the AMPure XP beads at 21°C for 30 min. Vortex thoroughly to ensure that the beads are properly mixed with the buffer.
  b. Prepare 80% ethanol solution in nuclease-free water.
  c. Aliquot 80 μL of AMPure XP beads into a V-bottom 96 well plate, according to the number of pooled genotyping PCR2 libraries.
  d. Add 100 μL of pooled genotyping PCR2 product to the beads (0.8:1 beads to DNA ratio) and pipette mix.
  e. Incubate for 5 min at 21°C.
  f. Place the plate on a magnetic stand and incubate for 2 min until the liquid is clear of beads.
  g. Carefully remove the supernatant.
  h. Wash the beads twice by adding 200 μL of 80% ethanol to each well, incubating for 30 sec and then removing and discarding the supernatant. After the second wash, remove any residual ethanol using 20 μL tips.
  i. Let the beads air-dry for 2–5 min. The beads are dry enough when the surface of the pellet changes from shiny to matt.
    **CRITICAL:** It is important to remove all residual ethanol but be careful not to over-dry the beads!
  j. Remove the plate from the magnet, resuspend beads in 50 μL EB buffer and mix thoroughly by pipetting.
  k. Incubate for 3 min to elute DNA.
  l. Place the plate back on the magnet and wait for 2–3 min for the supernatant to be completely clear of beads.
  m. Transfer the supernatant containing purified genotyping libraries to new LoBind microcentrifuge tubes. Take care not to transfer any beads.
    **Pause point:** The purified genotyping libraries can be stored at −20°C for at least two months.
77. Measure the concentration of each genotyping library using the Qubit dsDNA HS Assay Kit.
78. Check the size distribution of each genotyping library using a microcapillary assay such as Agilent Bioanalyzer or TapeStation (D1000 ScreenTape and Reagents). Check that each pool contains peaks at the expected size and record the average library size by choosing the region between 50 and 900 bp. Representative good quality libraries can be found in Figure 19. Troubleshooting 8 and 9.

79. Combine equimolar ratios of each pool to a fresh DNA LoBind 1.5 mL microcentrifuge tube (or equivalent), for pools that will be sequenced on the same sequencing run.
  a. When pooling, consider the average library size, the concentration, and the number of amplicons per pool. Use Table S4 as template.
  b. Dilute the final pool to 4 nM using Qubit and Bioanalyzer readouts.
80. Sequence the library (Figure 20) on an Illumina platform using the single-use sequencing primer aliquots that were prepared in preparation 5.
  ***Note:*** The choice of kit depends on the desired sequencing depth: we usually aim for 2,000 reads per cell per amplicon.
  **CRITICAL:** Make use of a PhiX spike-in (5%–10%), given the low genotyping library complexity! If PhiX is not used, the sequencing run is likely to fail.
  a. For the MiSeq use custom sequencing CS1 (read 1), CS2 (read 2), and CS2rc (index read) primers:
    i. Pierce ports 12, 13 and 14 containing Illumina sequencing primers with separate 1 mL tips. Aspirate the entire volumes into 3 tubes, labeled I12, I13, and I14.
    ii. Mix 10 μL of 100 μM CS1 with 10 μL of 100 μM CS2 to obtain 20 μL of CS1/CS2 50 μM (referred to as to “FL1”).
    iii. Mix 7 μL of FL1 with 200 μL of I12 and with 493 μL of HT1 buffer. Vortex and centrifuge briefly. Transfer the entire volume into port 18.
    iv. Mix 3.5 μL of CS2rc 100 μM with 200 μL of I13 and with 496.5 μL of HT1 buffer. Vortex and centrifuge briefly. Transfer the entire volume into port 19.
    v. Mix 7 μL of FL1 with 200 μL of I14 and with 493 μL of HT1 buffer. Vortex and centrifuge briefly. Transfer the entire volume into port 20.
    vi. Denature and dilute the 4 nM library from point 83a following the Illumina guidelines. Spike in 5%–10% of PhiX.
  b. For the NextSeq use custom sequencing LCS1 (read 1), CS2 (read 2), and CS2rc (index read) primers:
    i. Pierce ports 20, 21 and 22 containing Illumina sequencing primers with separate 1 mL tips. Aspirate the entire volumes into 3 tubes, labeled I20, I21, and I22.
    ii. Mix 6 μL of LCS1 100 μM primer with 800 μL of I20 and with 1194 μL of HT1. Vortex and centrifuge briefly. Transfer the entire volume into port 7.
    iii. Mix 6 μL of CS2 100 μM primer with 800 μL of I21 and with 1194 μL of HT1. Vortex and centrifuge briefly. Transfer the entire volume into port 8.
    iv. Mix 6 μL of CS2rc 100 μM primer with 1994 μL of HT1. Vortex and centrifuge briefly. Transfer the entire volume into port 9.
      ***Note:*** I22 is not required for sequencing PhiX and does not need to be added to the custom CS2rc index primer.
    v. Denature and dilute the 4 nM library from point 83a following the Illumina guidelines. Spike in 5%–10% of PhiX.
  c. For both platforms, set the following sequencing configuration: 151 cycles (read 1) + 10 cycles (index read) + 151 cycles (read 2).
    ***Note:*** CS1/LCS1 and CS2 primers will bind to CS1 and CS2 adapters inserted during PCR1, allowing the amplicon and plate barcodes to be sequenced. The CS2rc primer is the reverse complement of the CS2 adapter allowing the 10 bp cell index inserted during PCR2 to be sequenced.

## Expected outcomes

Whole-transcriptome 3′ biased library generation: after part 4, point 44, you should expect to generate cDNA library traces with the shape comparable to those in Figure 2. After bead purification and elution in ∼25 μL of EB buffer, we usually obtain 10-30 ng/μL of cDNA library per plate (190 cells). Note that these numbers apply to primary human bone marrow samples and may be influenced by both the RNA content of the cells sorted and by the PCR cycle number. After tagmentation and PCR, at part 5, point 55, the trace of the tagmented libraries should have a smooth shape comparable to the left graph in Figure 15. After bead purification and elution in ∼20 μL of EB buffer, we usually obtain 15-40 ng/μL of tagmented library per plate (190 cells). If sequencing on the NovaSeq, you should expect > 84% of bases > Q30. After running the pre-processing pipeline, you should ideally expect ∼80% or more wells passing QC filters (however, there can be considerable sample-to-sample variation in this regard, depending on sample quality). Successful human bone marrow hematopoietic stem and progenitor cells sequenced at a depth of 500,000–1,000,000 reads/cell on average display approximately: 350,000-750,000 reads assigned to genes; 6,500 unique genes detected per cell; 5% of reads mapping to the mitochondrial genome; 2% ERCC reads.

Single-cell genotyping library generation: after part 6, point 77, each genotyping library should contain 5–100 ng/μL of DNA. However, the yield depends on the number of loci amplified in each reaction. After part 6, point 78, for each library, you should observe bands of the expected size, (example in Figure 19). Additional low-intensity bands are not a problem if the strongest bands are the ones of the expected size. If multiple amplicons are present in the same mix, usually some bands will be stronger than others, which is usually also not a point of concern. If sequencing on the NextSeq, you should expect at least 85% of bases > Q30. In a good quality run, 90%–95% of cells that pass scRNA-seq QC should also be successfully genotyped.

## Quantification and statistical analysis

TARGET-seq+ yields three layers of data per single cell, which should be integrated for multimodal analysis.

1. FACS indexing of cell surface protein expression.
2. Targeted genotyping.
3. RNA sequencing.

Broadly, the analysis workflow follows these steps.

4. Analysis of FACS indexing data.
5. Analysis of single-cell genotyping data within each patient sample and integration with flow cytometry indexing data to generate metadata for the full dataset.
6. Pre-processing of transcriptome data.
7. Integration of the full dataset for downstream analysis.

Detailed instructions, scripts and an example dataset are available on GitHub (https://github.com/asgerjakobsen/TARGET-seq-plus). These scripts may be used to analyze TARGET-seq+ data from human HSPCs, but may be adapted for datasets using other cell types, FACS panels, etc.

### Analysis of FACS indexing data

Run the FACS_indexing_analysis.R script to gather cell surface immunophenotyping data from FACS index sorting files into a single table. From these data, we know the sample donor ID of each cell, which is important for defining which cells belong to the wild-type control sample and which belong to the test samples when performing downstream analysis. Using the cell surface immunofluorescence values, we can gate for positive and negative cells to define immunophenotypic populations.

### Analysis of targeted genotyping data

8. First, generate a demultiplexing file with details of the plate barcodes and single-cell barcodes used for each well in single-cell genotyping library preparation by running the 01_Make_genotyping_demultiplexing_file.R script.
9. We use the TARGET-seq analysis pipeline (https://github.com/albarmeira/TARGET-seq)^3^ to pre-process targeted single-cell genotyping data.
  a. Demultiplex the data into separate FASTQ files for each cell by running the GenoDemux_Fastq.sh script.
  b. Run the SCgenotype.pl script (https://github.com/albarmeira/TARGET-seq) to align reads to the genome, separate cDNA/gDNA amplicons and perform mpileup variant calling. The script generates summary tables of allelic counts for each mutation locus per cell (Figure 21). Troubleshooting 10, 14, 15, and 17.

**Figure 21:**
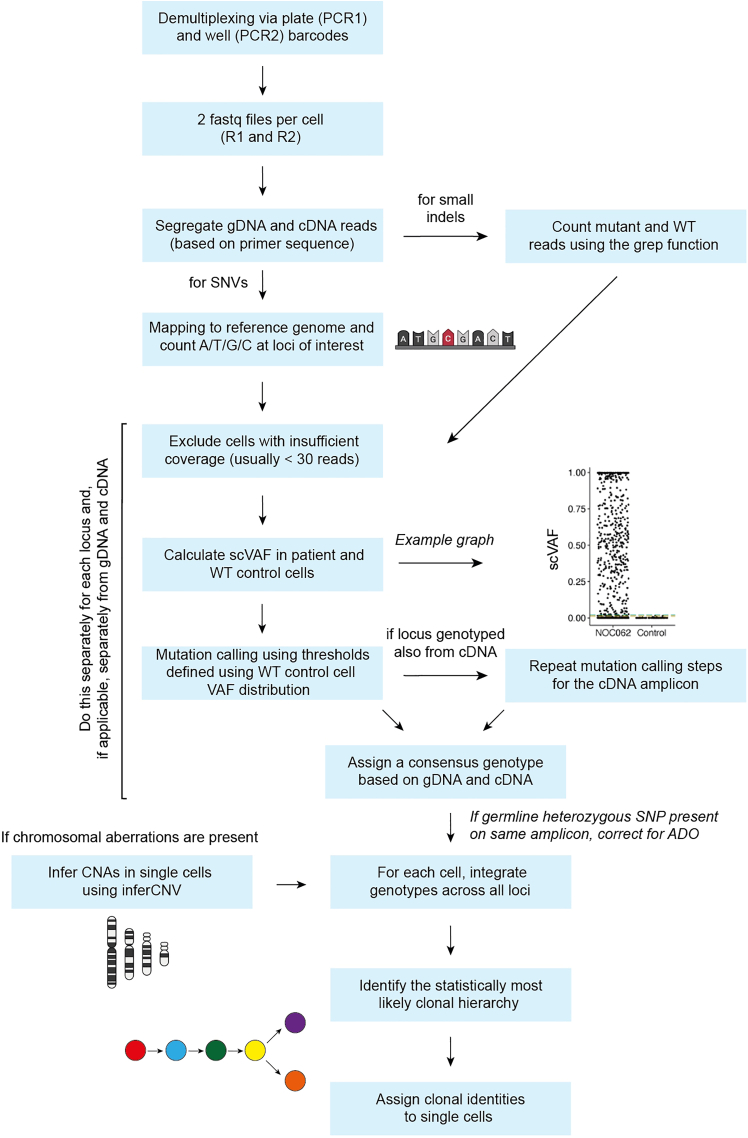
Computational workflow for single-cell genotyping analysis Note that the pre-processing modality differs between single-nucleotide variants (SNVs) and small insertions or deletions (indels). scVAF, single-cell VAF; CAN, copy number alteration; ADO, allelic drop-out; SNP, single-nucleotide polymorphism.
10. For indels, we use a separate pipeline, because these are often not called correctly by mpileup variant calling:
  a. Create FASTA files with the genotyping PCR1 primer sequences.
  b. Run the indel_mutation_grep.sh script. This takes demultiplexed FASTQ files as input and uses cutadapt to separate cDNA/gDNA amplicons based on the PCR1 primer sequences. It then uses fastq-grep to count the reads with the WT and mutant sequence in each cell. Use a minimal unique sequence for the grep function, e.g. 5 bp either side of the mutation. Depending on the site of the mutation, either the forward (R1) or the reverse (R2) sequencing reads (or both) should be used for allele counting.
  c. The resulting allele count files can be used in genotyping calling analysis in the same way as those from the TARGET-seq pipeline.
11. We then use the allelic counts at each locus of interest to assign a genotype to each cell (Figure 21). We provide two example scripts to illustrate these steps. Troubleshooting 16.
  a. Calculate the total coverage and variant allele frequency of the mutation within each single cell (scVAF) for each amplicon separately.
  b. Filter out cells with low coverage of the mutation. We base the minimum coverage threshold on the reads observed in empty no-template control wells (Figure 22).

**Figure 22:**
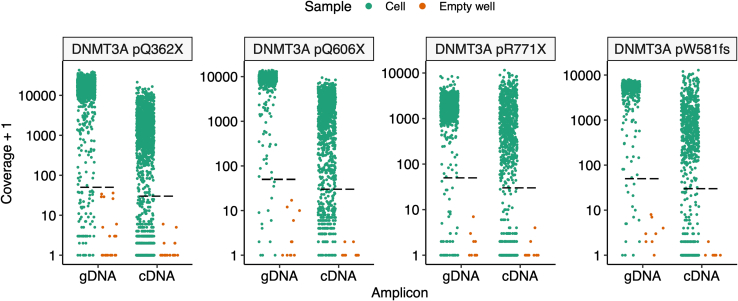
Controlling for cross-well contamination Plots of sequencing coverage across genotyping loci, comparing single cells (green) and empty wells (no-template controls; orange). The minimum coverage for calling genotypes was set at 50 reads for gDNA amplicons and 30 reads for cDNA amplicons (indicated by dashed lines). The loci shown are selected from those genotyped in our original paper.^1^
  c. Assign a genotype to each cell based on the scVAF of the mutation for each amplicon separately. The scVAF distribution of WT control cells (scVAF_WT-CTRL_) may be used to define the error rate at each locus and thereby set a scVAF threshold above which cells are called mutant (Figure 23A). We set two thresholds:
    i. We define a lower scVAF threshold, below which cells are called WT:
      scVAF threshold for calling a cell WT = mean(scVAF_WT-CTRL_) + 3∗SD(scVAF_WT-CTRL_).
    ii. We define an upper scVAF threshold, above which cells are called mutant:
      scVAF threshold for calling a cell mutant = mean(scVAF_WT-CTRL_) + 3∗SD(scVAF_WT-CTRL_) + 0.01.
    iii. Cells with scVAF between these thresholds are called undetermined.

**Figure 23:**
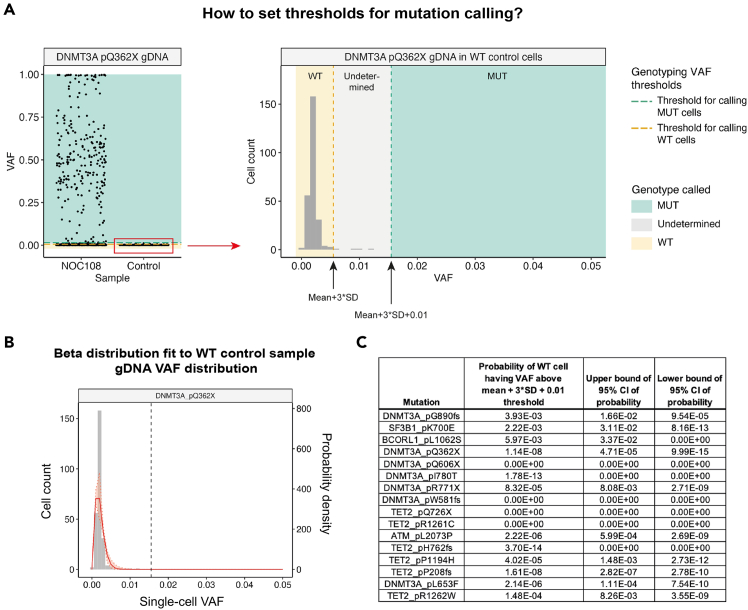
Setting the scVAF thresholds for mutation calling in single cells (A) Left plot: Plot of the scVAF distribution for the *DNMT3A* pQ362X mutation (genotyped in our original paper^1^), comparing cells from a mutant sample (NOC108, left of the graph) versus the WT control sample (right of the graph). Right plot: To better visualize the scVAF thresholds, a histogram of the VAF distribution in cells from the WT control sample at scVAF 0–0.05 is plotted. Cells below the lower threshold (mean + 3∗SD, orange) are called WT; cells between the two thresholds (gray) are called undetermined; cells above the upper threshold (mean + 3∗SD + 0.01, green) are called mutant. (B) Example of beta distribution fit to the scVAF distribution in WT-control cells for the *DNMT3A* pQ362X mutation. By bootstrapping, the probability density distribution can be used to estimate the probability that a WT cell has a scVAF above a given threshold. The gray bars show the distribution of cells (left y-axis, cell number) with different scVAFs (x-axis). The solid red line shows the estimate of the probability density distribution (right y-axis shows probability density). The shaded red area shows the 95% confidence interval of the probability density distribution (estimated by bootstrap). Vertical dashed lines show the threshold for calling a cell heterozygous mutant (mean + 3∗SD + 0.01). (C) Probability of a WT cell having a measured scVAF above the mean + 3∗SD + 0.01 threshold for 16 mutations genotyped in our original paper.^1^ Upper and lower bounds of the 95% bootstrap confidence interval of the probability estimate are also shown.
  d. Call a consensus genotype by integrating the gDNA and cDNA calls (Figure 21).
    ***Note:*** In single-cell genotyping a distribution of VAFs is observed in heterozygous mutant cells, ranging from approximately 1% (0.01) to 99% (0.99), due to unequal amplification of the original alleles during PCR. Furthermore, the scVAF of wild-type cells is commonly not equal to 0 due to noise generated during PCR amplification and next-generation sequencing. These sources of noise need to be accounted for when calling genotypes. To define the appropriate scVAF thresholds for mutation calling, we use data from WT control cells where the genotype is known *a priori* to determine the error rate at each locus. We have benchmarked this strategy for defining genotyping thresholds across 16 loci (Figures 23B and 23C). We have found that the probability of WT cells having a scVAF measurement above the threshold used to call cells heterozygous mutant (i.e. false positive mutant calls) was consistently below 0.006.
    ***Note:*** Cells that appear WT for a mutation can either be genuinely WT or there may have been allelic dropout (ADO) of the allele harboring the mutation (Figure 24A), and it is not normally possible to distinguish these two scenarios. However, when a heterozygous germline single nucleotide polymorphism (SNP) is present within the genotyping amplicon, this may be used to determine whether both alleles have been sampled in each cell (Figure 24B). Variant calling at the SNP locus is performed in the same way as for the mutations (Figure 24C). By phasing the SNP alleles with the mutation (Figure 24B), it is possible to identify cells in which the mutant allele has dropped out and thus exclude these from the analysis due to the uncertainty regarding their genotype. This also makes it possible to calculate the ADO rate.

**Figure 24:**
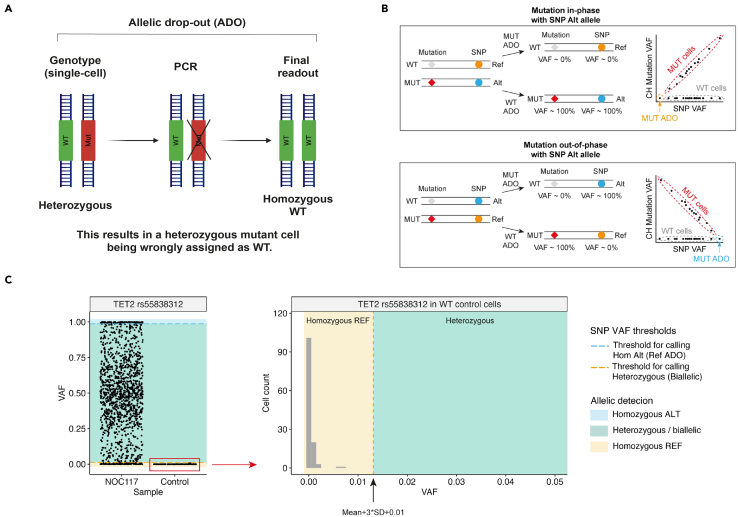
Allelic drop-out controls using germline heterozygous SNPs (A) Schematics of allelic drop-out. (B) Schematic overview of how germline heterozygous SNPs may be phased with mutations in the same genotyping amplicon. Top, when the SNP Alternate (Alt) allele is in-phase with the mutation (i.e. they are located on the same allele), the single-cell variant allele frequency (scVAF) of the mutation and the SNP are correlated. Cells where there was ADO of the mutant allele will have a scVAF close to 0% for both the mutation and the SNP. Bottom, when the SNP Alt allele is out-of-phase with the mutation (i.e. they are located on opposite alleles), the scVAF of the mutation and the SNP are anti-correlated. Cells where there was ADO of the mutant allele will have a scVAF of ∼0% for the mutation but a scVAF of ∼100% for the SNP Alt allele. Plot adapted from our original paper.^1^ (C) Plot of the scVAF distribution for the TET2 rs55838312 germline SNP, comparing cells from a heterozygous sample (NOC117, left of the graph) versus the WT control sample (right of the graph). Cells below threshold (mean + 3∗SD + 0.01, orange) are called Homozygous REF; cells between the two thresholds (green) are called heterozygous (Biallelic detection); cells above the upper threshold (blue, at the very top of the graph) are called Homozygous ALT.
12. Once genotypes have been called for each mutation locus separately, integrate the genotypes across all mutations within each sample to assign clonal identities to each cell (Figure 21).
  a. We use infSCITE^15^ to identify the statistically most likely clonal hierarchy.
  b. Assign clonal identities to each cell. The 04_Clone_assignment_NOC131.R script provides an example of how to do this, with example data from a sample with two independent clones.

***Optional:*** The identification of the most likely clonal hierarchy can be done using alternative algorithms. In case of difficult/ambiguous clonal structures, it may be worth comparing the results from more than one algorithm.

13. Integrate the FACS indexing and single-cell genotyping data to make a metadata file for multi-omic analysis by running the 05_Integrate_metadata.R script.

### Pre-processing of transcriptome data

After sequencing, reads should be demultiplexed by providing a standard Illumina sample sheet containing the plate identifiers and the i5/i7 index combinations used for each plate, or by running bcl2fastq.

After plate demultiplexing, RNA sequencing data can be pre-processed using a custom pipeline available on GitHub: https://github.com/asgerjakobsen/TARGET-seq-plus-RNA. The pipeline processes the data in the following steps.

14. Demultiplex reads from each plate into single cells using the 14 bp oligo(dT) cell barcodes sequenced in Read 1. This generates individual FASTQ files with single-ended cDNA reads corresponding to each single cell.
15. Concurrently, trim the cDNA reads for poly(A) tails, Nextera adapters and low-quality reads.
16. Map the reads to the genome reference assembly using STARsolo. We use the hg38 human reference genome with ERCC92 transcripts.
17. Concurrently, perform gene feature counting using STARsolo to generate a counts matrix.

### Integration of the full dataset for downstream analysis

Integration of transcriptome data with genotyping and FACS indexing data relies on shared cell identifiers. The transcriptome cell barcodes in the outputs from the pre-processing pipeline are derived from the combination of libraries with unique i5/i7 plate indexes and oligo(dT) cell barcodes. These need to be reformatted to match those in the other metadata. Run the Reformat_counts_matrix.R script to join the transcriptome cell barcodes to the descriptive cell IDs that match those in the other metadata. The script then reformats the outputs from STARsolo to add gene names and cell IDs to the transcriptome counts matrix.

The resulting counts matrix can be used with the integrated metadata file in single-cell genomics analysis packages for downstream analysis. Troubleshooting 18.

## Limitations

The main limitation of TARGET-seq+ is limited cell throughput compared to droplet-based approaches.^16^^,^^17^^,^^18^^,^^19^ In terms of cell numbers, it is feasible to apply TARGET-seq+ to process 5000 cells in 3-4 weeks, at a cost of ∼3–4$ per cell. However, most sorted cells pass RNA-seq QC filters and are successfully genotyped, partially compensating for limited throughput. Of note, while droplet-based methods^16^^,^^17^^,^^18^^,^^19^ achieve higher throughput, these methods currently suffer from (a) much lower genotyping sensitivity and higher allelic drop-out rates, due to the reliance on genotyping from cDNA; and (b) lower transcriptome library complexity, which may be problematic when lowly expressed genes are analyzed. Of note, the time required for the protocol increases if many mutations are genotyped, given that the number of genotyping PCR1 and PCR2 reactions per cell increases. Hence, in somatically very complex tumors/tissues, TARGET-seq+ analysis needs to be limited to a tractable number of key mutations (we would suggest up to 12).

Second, similar to other methods for integrating single-cell genotyping to RNA-seq or chromatin accessibility,^8^^,^^16^^,^^17^^,^^18^^,^^19^^,^^20^ TARGET-seq+ relies on prior knowledge of mutations previously detected in the same samples by bulk sequencing. Hence, a portion of the initial sample needs to be used for driver mutation discovery prior to TARGET-seq+.

Third, certain steps like single-cell sorting and cDNA library generation from single cells are extremely time-sensitive (due to RNA degradation). We would thus suggest new users to be trained first by an experienced molecular biologist prior to the execution of the method.

Finally, TARGET-seq+ currently does not allow for simultaneous capture of readouts like chromatin accessibility. Other approaches like GTAC^8^ (plate-based) or GoTCha^20^ (droplet-based) capture single-cell genotype and chromatin accessibility but fail to capture gene expression. Hence, to gain a deeper understanding of clone-specific gene regulation, methods for clone-aware scRNA-seq and scATAC-seq need to be performed separately, followed by computational integration of the two datasets.

## Troubleshooting

We list here the most recurrent problems we encountered while deploying TARGET-seq+, with potential solutions. For useful experience from other users and potential additional problems not listed here, we advise to read the troubleshooting section in the original TARGET-seq protocol.^3^

### Problem 1

The QC by capillary electrophoresis and Qubit shows no cDNA libraries or a very low cDNA yield (part 4, points 44–45).

### Potential solution

Provided that all primers and reagents have been properly added, there are several potential reasons and solutions for this problem.

• The single-cell FACS sort failed (part 2, points 21–26). Make sure that the sorter is properly calibrated for sorting into 384-well plates. After every 5^th^ sorted plate, re-do the calibration test to make sure the calibration is still appropriate. Additional tips related to single-cell sorting can be found in our prior protocol.^9^
• RNA degraded prior to RT due to the single-cell sort taking too long. Make sure that cell sorting into a plate lasts no longer than 15 min from the time when the first well is sorted (part 2, point 25). Snap freeze the plate immediately after the sort (part 2, point 26). Moreover, the RNA can also degrade if the time interval between protease inactivation and RT took too long. Make sure to start the RT program within 5 min from when protease inactivation is finished (part 3, point 33).
• RNA degraded prior to RT due to RNase contamination. Prior to the PCR, work strictly in a pre-PCR environment, always clean the pipettes and the working surfaces with a cleaning agent to remove RNases, work with RNase-free tips, and always use nuclease-free water. Moreover, always keep the lysis buffer cold so the protease does not degrade the RNase inhibitor (Parts 1 and 3).
• The RT step failed. The TSO is the most sensitive component of the RT mix and should be handled with care. After reconstitution, make single-use TSO aliquots (∼72 μL for 4 plates) and keep them at −80°C for a maximum of 2 months (materials and equipment). Prior to RT, keep the TSO on dry ice and only thaw it while preparing the RT mix. After adding the TSO to the mix, keep the RT mix cold. Also, avoid vortexing the TSO (part 3, point 32).
• The RT step took too long. Make sure that part 3, point 33 does not take more than 5–6 min.
• The number of PCR cycles is insufficient (part 3, point 39). Make sure you optimize the number of PCR cycles for the tissue of interest in preparation 1.

### Problem 2

The QC by capillary electrophoresis shows the cDNA library size distribution is skewed towards lower ranges than those shown in Figure 2 (part 4, point 44).

### Potential solution

This is indicative of RNA degradation. Please see the potential solutions to problem 1 for advice on how to avoid RNA degradation. If all the steps were performed according to best practice and RNA degradation is still an issue, we advise ordering fresh reagents. Poor cell viability may also be a cause of RNA degradation (part 2, point 12a).

### Problem 3

The QC by capillary electrophoresis shows cDNA libraries displaying many prominent spikes and potentially skewed to lower ranges than those shown in Figure 2 (part 4, point 44).

### Potential solution

This is usually a sign of concatemer formation.

• Make sure your genotyping primers are not interfering with cDNA amplification. Check your testing results from preparation 4 to make sure no genotyping primers were associated with concatemer formation (Figure 6B).
• Alternatively, this could by sign of TSO concatemers which may be independent of genotyping primers. Make sure the TSO has been stored appropriately, and that you are not using a TSO older than 6 months (materials and equipment). To minimize the risk of TSO concatemers, we advise ordering a biotinylated TSO.

### Problem 4

The QC by capillary electrophoresis shows no cDNA libraries and just two prominent peaks around 600 bp and 1,300 bp (part 4, point 44).

### Potential solution

These peaks may come from the ERCC spike-in RNA. Double-check the concentration of ERCC in the ERCC stock and in the lysis buffer (materials and equipment and part 1, point 1).

### Problem 5

The QC by capillary electrophoresis shows tagmented cDNA library with periodic spikes (‘hedgehog’ pattern, right panel in Figure 15; part 5, point 55) despite no major problems were detected with cDNA libraries.

### Potential solution

Sometimes, TSO concatemers may become obvious only after tagmentation and PCR. Please refer to our potential solution to problems 1 and 3 to avoid TSO concatemers.

### Problem 6

The QC by capillary electrophoresis shows under-tagmented cDNA libraries (tagmented libraries skewed to higher ranges compared to the left panel in Figure 15; part 5, point 55).

### Potential solution

• You may be loading excessive cDNA into the tagmentation. Re-measure the cDNA concentration with Qubit and, additionally, using the Bioanalyzer region settings (part 4, points 44–45).
• If there is contamination of very large fragments in your cDNA, this may also lead to apparent under-tagmentation. Try to remove aberrantly large fragments using size exclusion via bead clean-up but pay attention not to remove your cDNA fragments (part 4, point 43).
• If the problem persists, try loading less cDNA than recommended (part 5, point 46).
• Also, make sure the tagmentation time is set to no less than 10 min (part 5, point 50).
• Finally, use the Tn5 enzyme within its expiration date and make sure you are adding sufficient enzyme to the reaction (part 5, point 47).

### Problem 7

The QC by capillary electrophoresis shows over-tagmented cDNA libraries (tagmented libraries skewed to lower ranges compared to the left panel in Figure 15; part 5, point 55).

### Potential solution

• You may be loading insufficient cDNA into the tagmentation. Re-measure the concentration with Qubit and, additionally, using the Bioanalyzer region settings (part 4, points 44–45).
• It is also possible that there is a problem with the neutralization buffer. Use the buffer within the expiration date. Add the buffer as soon as tagmentation is finished and pipette-mix well. If you are using a homemade SDS dilution, make sure the SDS is diluted to the right concentration (part 5, point 51).
• Make sure you start tagmentation as soon as you combine the tagmentation mix with the cDNA. Do not let the cDNA/tagmentation mix sit for too long prior to tagmentation (part 5, point 50). If you have many samples, use a multichannel pipette to speed up the process (part 5, point 49).
• If the problem persists, try loading more cDNA than recommended (part 5, point 46).
• Also, make sure the tagmentation time is set to no more than 10 min (part 5, point 50).

### Problem 8

The QC of genotyping libraries by capillary electrophoresis shows no or very weak genotyping bands, accompanied by low amplicon yield as measured by Qubit (part 6, points 77–78).

### Potential solution

This is usually due to inefficient pre-amplification of the amplicon(s) in the RT-PCR reaction due to suboptimal genotyping primers. Make sure to validate all pre-amplification and nested genotyping primers (preparation 2, 3, and 4) prior to usage. If there are signs of non-specific amplification (Figures 4 and 7), redesign the primers to minimize off-target binding and 3′ self-complementarity. For long-term storage, keep all genotyping primers frozen in TE buffer to avoid degradation.

### Problem 9

The QC of genotyping libraries by capillary electrophoresis shows a second peak in close proximity to the expected peak, ∼50 bp shorter than the expected peak (part 6, point 78).

### Potential solution

The unexpected shorter peak likely corresponds to genotyping amplicons that are not barcoded by PCR2 primers. This usually occurs if there is excessive concentration of the nested PCR1 genotyping primers, which then compete with the PCR2 primers for the same amplicon when carried over into the PCR2 reaction. Check the concentration of nested PCR1 genotyping primers used in PCR1 (part 6, point 59) and make sure to use a sufficient concentration of PCR2 barcoded primers (part 6, point 67). If the problem persists, consider reducing the concentration of nested PCR1 genotyping primers used in PCR1 (part 6, point 59).

### Problem 10

Insufficient coverage of the genotyping locus/loci in most cells despite good scRNA-seq libraries (quantification and statistical analysis).

### Potential solution

This indicates that the single-cell sort worked, but there is a problem with single-cell genotyping.

• Make sure you tested all genotyping primers prior to the experiment (preparation 2, 3, and 4).
• Go back to single-cell primer testing for the problematic loci and make sure that the primers yielded strong amplicons in single cells (preparation 4).
• If all testing results seem fine and there is no apparent reason for genotyping failure, it may be possible that the sample of interest contains a heterozygous or homozygous germline SNP within the sequence bound by the pre-amplification or nested primer; this would explain why the primers yielded good results in testing but poor results in the sample of interest. It may be useful to check for potential SNPs in bulk NGS data prior to primer design.

### Problem 11

Failed sequencing run for single-cell genotyping or transcriptome libraries despite good QC results by capillary electrophoresis (part 5, point 58 and part 6, point 80).

### Potential solution

• In our experience, the most common reason for failed sequencing of single-cell genotyping libraries is the low complexity of the library (i.e. a low number of unique fragments). This can happen especially if you are only sequencing a small number of different amplicons. Make sure that enough PhiX was loaded for low complexity libraries. For very low-complexity libraries, you may need to increase the PhiX to 20%.
• Failed sequencing of transcriptome libraries may arise from low complexity due to prominent TSO concatemers (Figure 15), or sequencing into the poly(A)-tail in Read 1. Check the capillary electrophoresis results for signs of concatemers which may appear as episodic spikes in the fragment size distribution (Figure 15). Ensure the sequencing configuration is set to 15 cycles in Read 1 (part 5, point 58).
• Sequencing primers: (a) make sure that sequencing primer sequences are correct; (b) make sure to use single-use aliquots of sequencing primers, stored at −20°C (materials and equipment); (c) make sure that the correct primers are loaded in the correct ports of the sequencing kit (part 6, point 80).
• In case of failed single-cell genotyping libraries, make sure that the genotyping amplicons are not shorter than the number of cycles used for sequencing. This would cause sequencing of the CS adapters which may lead to extremely low complexity at later cycles.
• Less likely, there may also be a problem with the P5/P7 sequencing adapters. Make sure that the i7/S5 and PCR2 primers have been stored correctly.

### Problem 12

Lower sequencing yield than expected (i.e. under-clustering; part 5, point 58 and part 6, point 80).

### Potential solution

• This is usually caused by the overestimation of the concentration of fragments which can be sequenced (i.e. which have intact P5/P7 adapters). Although we recommend estimating the molarity by a combined Qubit and Bioanalyzer readout (part 5, point 57 and part 6, point 79), this method works accurately only if most of the fragments in your library have an intact P5/P7. For a more accurate estimate of the concentration of fragments which can be sequenced, alternative methods like the KAPA Library Quantification Kit for Illumina Platforms can be used.
• Moreover, we recommend using the Bioanalyzer or the Fragment Analyzer instead of a Tapestation system for the final library quantification (to obtain 4 nM libraries; part 5, point 57 and part 6, point 79).

### Problem 13

Unable to design working genotyping primers (preparation 4).

### Potential solution

Some loci are complicated to amplify, usually due to a high GC content.

• Search in the literature for potential primers designed for other purposes; test these primers in TARGET-seq+ (preparation 4).
• You may try to extend the region you are amplifying with the pre-amplification primers. Although we recommend designing amplicons < 900 bp, it may be possible to have efficient pre-amplification with larger amplicons, due to the elongation time of the first PCR being 6 min (preparation 2).
• You may also use the same primers for pre-amplification and PCR1 steps. Note that, if the distance from the beginning of the primer and the mutation is > 140 bp, you may need to perform more sequencing cycles and sequence the problematic amplicon separately.
• If multiple mutations are present within the sample and one of these is very challenging to amplify, try to infer from bulk NGS data whether some mutations are clonal to the problematic mutation. In those cases, genotyping these mutations may serve as a proxy to detect the clone of interest (note, however, that this is an indirect method).

### Problem 14

Sequencing of both libraries reveals many empty wells (quantification and statistical analysis).

### Potential solution

This indicates a problem with the single-cell FACS sort (part 2, points 21–26). Make sure to calibrate the sorter appropriately for sorting into 384-well plates (refer to our potential solution to problem 1). Moreover, ensure your FACS gates are set so as not to sort debris or dead/dying cells.

### Problem 15

Sequencing of both libraries reveals excessive sequencing reads in empty control wells (quantification and statistical analysis).

### Potential solution

This points to cross-well contamination in these plates. We recommend not using data from these plates, as it will introduce technical errors (WT control cells appear as mutant, inaccurate differential gene expression, etc.). To minimize cross-well contamination.

• Take great care when unsealing the plates. Hold the plates firmly against the bench while slowly removing the plastic or aluminum seals.
• Always centrifuge the plates as recommended, prior to unsealing them.
• Take great care when removing the metallic plate holders for the Mosquito; sometimes, they can stick to the plastic on the plate, making it challenging to remove them.

### Problem 16

The genotyping locus of interest shows only WT reads, whereas a significant proportion of mutant cells is expected (quantification and statistical analysis).

### Potential solution

• If you exclude that the discrepancy is due to a different cell composition, it is likely you may be using the wrong coordinates for read counting in the pre-processing genotyping pipeline. Double check that the coordinate for the nucleotide of interest is correct (quantification and statistical analysis).
• This could also happen when genotyping small indels, as these are challenging to detect with the pre-processing pipeline. Try using our alternative strategy of counting indel reads (quantification and statistical analysis).
• Less likely, it is possible that there is a germline heterozygous SNP in-phase with the mutation, which is located exactly in the sequence recognized by the primer. This would lead to preferential amplification of the WT allele. Check the bulk sequencing data for potential presence of SNPs.

### Problem 17

The genotyping locus of interest displays 0 allelic counts, although QC by capillary electrophoresis indicated successful single-cell genotyping library preparation (quantification and statistical analysis).

### Potential solution

It is likely that the nested primer sequence used for the pre-processing pipeline is wrong. Double-check the sequences correspond to the primers used for PCR1 (quantification and statistical analysis).

### Problem 18

scRNA-seq shows considerable batch effects between plates, even if cells from the same sample are sorted onto different plates (quantification and statistical analysis).

### Potential solution

This is likely technical in nature, as we do not usually observe prominent plate-related batch effects. Try processing the different plates at the same time, using the same batch of reagents across plates, and making sure that experimental procedures are consistent (for example, the timing of the sort or the RT).

## Resource availability

### Lead contact

Further information and requests for resources and reagents should be directed to and will be fulfilled by the lead contact, N. Asger Jakobsen (asger.jakobsen@imm.ox.ac.uk).

### Technical contact

Technical questions on executing this protocol should be directed to and will be answered by the technical contacts, N. Asger Jakobsen (asger.jakobsen@imm.ox.ac.uk) and Sven Turkalj (sven_turkalj@dfci.harvard.edu).

### Materials availability

The list of previously validated target-specific genotyping primers^1^^,^^10^ can be found in Table S1. The list of barcoded oligo(dT) primers and i5 indices can be found in Table S2.

### Data and code availability

The code for TARGET-seq+ single-cell FACS-indexing and genotyping analysis can be accessed at https://github.com/asgerjakobsen/TARGET-seq-plus and is also provided in an open access disposition at Zenodo: https://doi.org/10.5281/zenodo.14867073. The Perl script used for single-cell genotyping data pre-processing was published previously^2^ and is available at the respective GitHub page. The DOI is listed in the key resources table. The pipeline used for scRNA-seq data pre-processing can be accessed at https://github.com/asgerjakobsen/TARGET-seq-plus-RNA and is also provided in an open access disposition at Zenodo: https://doi.org/10.5281/zenodo.14867075. Raw single-cell genotyping data^1^ used to generate some of the figures in this protocol are available at European Genome-Phenome Archive (EGA: EGAS00001007358), and processed data are available through Figshare (Figshare: https://doi.org/10.25446/oxford.23576421).

## Acknowledgments

P.V. acknowledges funding from the 10.13039/501100000265Medical Research Council Molecular Haematology Unit Programme Grant (MC_UU_00029/8), 10.13039/501100015570Blood Cancer UK Programme Continuity Grant 13008, 10.13039/100014461NIHR Senior Fellowship, and the Oxford BRC Haematology Theme. N.A.J. was supported by a 10.13039/501100000265Medical Research Council and 10.13039/100015763Leukaemia UK Clinical Research Training Fellowship (MR/R002258/1), MRC DTP Supplementary Funding 2021, and a 10.13039/501100000265Medical Research Council Molecular Haematology Unit Programme Grant (MC_UU_00029/2). S.T. was supported by the Scatcherd European Scholarship in partnership with the 10.13039/501100000265Medical Research Council/10.13039/501100021079Radcliffe Department of Medicine and the Clarendon Fund. S.T. also acknowledges the MRC DTP Supplementary Funding 2024. The authors thank Dr. Alba Rodriguez-Meira and Dr. Bilyana Stoilova for insightful discussions. The authors also acknowledge the MRC WIMM Flow Cytometry Facility and Single Cell Facility. Figures, or part of Figures 1, 3, 5, 8, 9, 10, 11, 12, 13, 14, 16, 17, 18, 20, 21, and 24, as well as the graphical abstract, were created using BioRender.

## Author contributions

Conceptualization, N.A.J. and P.V.; methodology, N.A.J. and S.T.; investigation, N.A.J. and S.T.; formal analysis, N.A.J. and S.T.; visualization, N.A.J. and S.T.; supervision, P.V.; writing – original draft, N.A.J. and S.T.; writing – review and editing, all authors.

## Declaration of interests

P.V. is the co-founder and interim CSO and on the board of Yellowstone Bioscience and is on the SAB of Auron Therapeutics.

## Supplemental information

Table S1. Previously validated pre-amplification and corresponding nested genotyping primer pairs, related to Preparation 2–4

Table S2. Oligo(dT) and custom i5 barcodes, related to Part 1 and 5

Table S3. Antibody mixes used to stain human bone marrow MNCs, related to Part 2

Table S4. Library pooling templates, related to Part 5 and 6

Table S5. Template for design of barcoded nested genotyping primers, related to Part 6

Table S6. Template for genotyping PCR1 planning, related to Part 6
